# An enhanced classification system of various rice plant diseases based on multi-level handcrafted feature extraction technique

**DOI:** 10.1038/s41598-024-81143-1

**Published:** 2024-12-23

**Authors:** Yasmin M. Alsakar, Nehal A. Sakr, Mohammed Elmogy

**Affiliations:** Information Technology Department, Faculty of Computers and Information, Mansoura University, Mansoura, 35516 Egypt

**Keywords:** Rice leaf diseases, Feature extraction, Handcrafted, Deep learning, Classification, Electrical and electronic engineering, Computer science, Information technology

## Abstract

The rice plant is one of the most significant crops in the world, and it suffers from various diseases. The traditional methods for rice disease detection are complex and time-consuming, mainly depending on the expert’s experience. The explosive growth in image processing, computer vision, and deep learning techniques provides effective and innovative agriculture solutions for automatically detecting and classifying these diseases. Moreover, more information can be extracted from the input images due to different feature extraction techniques. This paper proposes a new system for detecting and classifying rice plant leaf diseases by fusing different features, including color texture with Local Binary Pattern (LBP) and color features with Color Correlogram (CC). The proposed system consists of five stages. First, input images acquire RGB images of rice plants. Second, image preprocessing applies data augmentation to solve imbalanced problems, and logarithmic transformation enhancement to handle illumination problems has been applied. Third, the features extraction stage is responsible for extracting color features using CC and color texture features using multi-level multi-channel local binary pattern (MCLBP). Fourth, the feature fusion stage provides complementary and discriminative information by concatenating the two types of features. Finally, the rice image classification stage has been applied using a one-against-all support vector machine (SVM). The proposed system has been evaluated on three benchmark datasets with six classes: Blast (BL), Bacterial Leaf Blight (BLB), Brown Spot (BS), Tungro (TU), Sheath Blight (SB), and Leaf Smut (LS) have been used. Rice Leaf Diseases First Dataset, Second Dataset, and Third Dataset achieved maximum accuracy of 99.53%, 99.4%, and 99.14%, respectively, with processing time from $100(\pm 17)ms$. Hence, the proposed system has achieved promising results compared to other state-of-the-art approaches.

## Introduction

Agriculture has a vital role for humans in many domains, such as livelihoods and food production, besides economic development. Rice is the most significant staple food worldwide^1,2^. The rising world population is predicted to increase the demand for rice. For over three billion people, rice is the primary food of choice^3^. A total of 15% of areas of agricultural farms worldwide have been utilized for rice farming^4^. Many losses affect the country’s gross domestic product (GDP) in agriculture, especially rice plants. These losses decrease plant growth and cause yield reduction. Hence, it is crucial to enhance rice production.

Many biotic or abiotic diseases affect the amount and quality of rice productivity. Microorganisms, such as viruses, fungi, amoeba, and bacteria in many plants, cause biotic diseases. Non-living organisms, such as burning chemicals, hair, and weather conditions, cause abiotic diseases. In contrast, abiotic diseases are non-infectious, dangerous, and preventable. There are many diseases related to rice, such as blast (BL), bacterial leaf blight (BLB), brown spot (BS), tungro (TU), and sheath blight (SB). Concentrating on rice plants, a discussion of various types of rice plant diseases is presented in the following subsection.

### Rice plant diseases

**Fig. 1:**
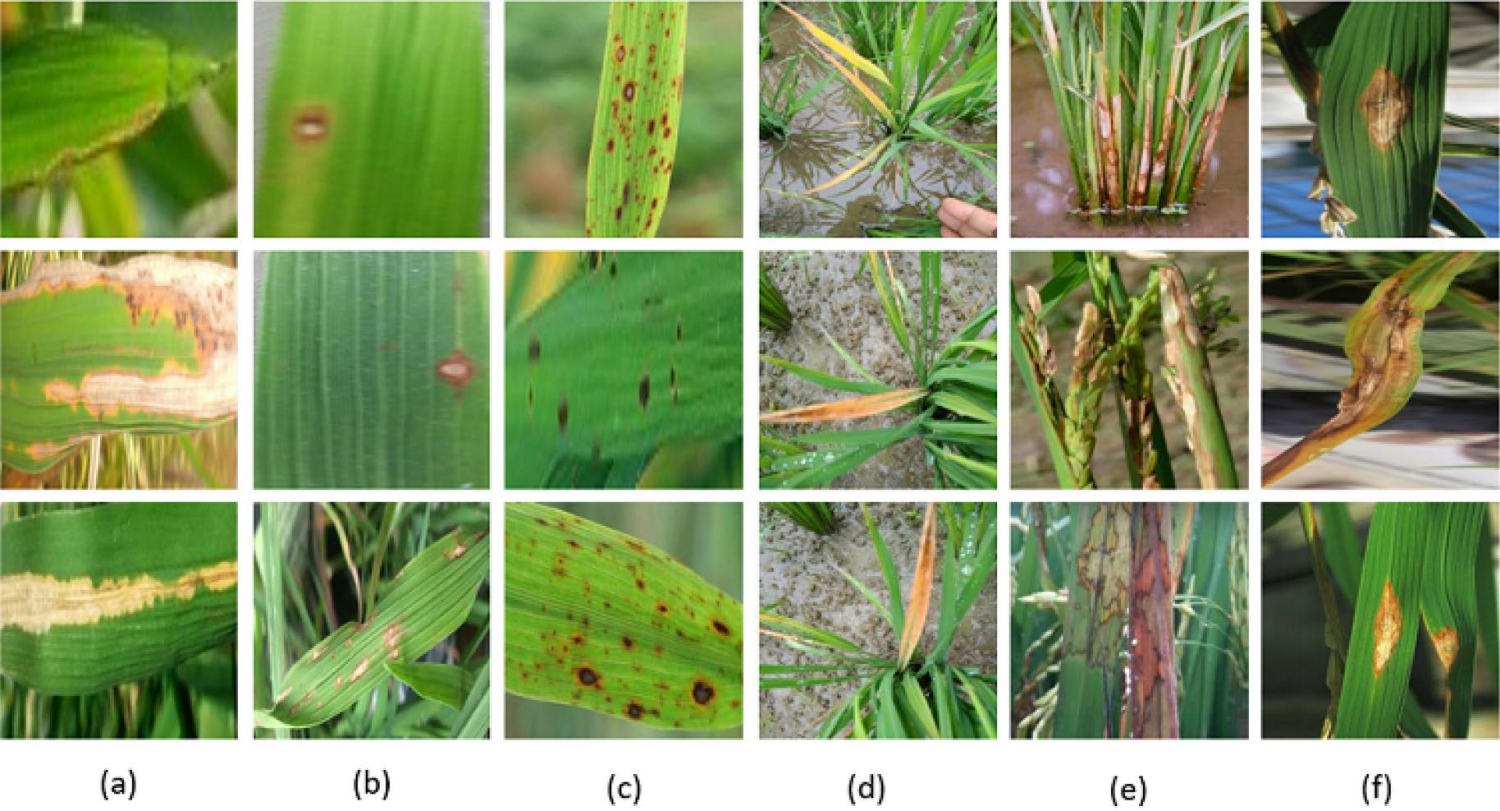
Examples of the rice leaf diseases taken from the used datasets: (**a**) BLB, (**b**) BL, (**c**) BS, (**d**) TU, (**e**) SB, and (**f**) LS.

Various pathogenic microorganisms destroy rice plants’ leaves, leading to many losses in the rice fields  ^5^. These microorganisms, such as fungi, viruses, bacteria, and amoebain many plants, cause biotic diseases. These diseases reduce the quantity and quality of rice products. The most common rice diseases are BLB, BL, BS, TU, and SB, which are discussed below. Fig. 1 illustrated some examples of the images of rice leaf diseases.

- **Bacterial Leaf Blight (BLB):** It is the most dangerous rice leaf disease caused by “Xanthomonas oryzae” bacteria. The Japanese farmers first identified this disease in 1884^6^. It is widespread in various regions of Africa, northern Australia, and the United States^7^. The leaf affected by this disease has a gray-green, takes the color of straw (yellow), and finally dies after withering. The lesions have sharp borders and infect the bottom.
- **Blast (BL):** It is the most harmful disease that affects rice production, and this causes a threat to food safety. Magnaporthe oryzae causes it^8^. It is a fungal disease. The first symptom is a change from white to patches of grey-green color that are concise (pivot-shaped), after becoming dark-red color and finally dark-brown edges. This disease has fewer crystal shapes with sharp ends and big cores.
- **Brown Spot (BS):** It is a damaging and common rice leaf disease caused by fungi^9^. Rice diseases have many large spots that harm and affect the leaf. The round, small leaf’s color turns into purple-brown dark brown lesions. These can be discovered early on, producing lesions that turn into elliptical shapes with a light brown color to gray core with a reddish-brown perimeter caused by the fungi’s blights.
- **Tungro (TU):** It is a very dangerous rice disease because of the virus^10^. It is found because the two viruses mix and are increased by green leafhoppers. It causes the slow development of rice plants, leaf discoloration, partially or sterile-filled grains, and fewer tillers. This disease affected cultivated rice, wild rice cousins, and other grassy weeds.
- **Sheath Blight (SB):** It is a widespread and dangerous rice disease caused by soilborne fungus. This disease is named ’snakeskin disease’ and ’rotten foot stalk’^11^. Firstly, the Sheath blight symptom is a water-soaked lesion on leaves. After two or three days, this lesion has a grayish-white center surrounded by a dark color. After BL disease, this is the second most frequent rice disease and the most significant commercially^12^.
- **Leaf Smut (LS):** Leaf smut (LS) is caused by a fungus named Entyloma oryzae^13^. Even though it is not a serious disease, it can lead to other diseases by making an environment favorable to other fungi’s growth. This disease’s symptoms are tiny spots scattered through the leaf in a non-uniform shape. The lesions of leaf smut are reddish brown circular.

Various rice diseases affect the growth and productivity of crops, causing many ecological and economic problems. For this reason, it is very significant to identify these diseases. Correcting and identifying rice diseases can be performed manually or using computer-aided systems. Plant pathologists perform manual predication, which can be lengthy, tiresome, and expensive and may cause fatigue errors. Therefore, new advancements like machine learning and deep learning-based artificial intelligence have great attention in computer vision algorithms that are used in plant disease identification^14–17^.

Computer-aided rice leaf disease detection and classification systems face many challenges. These challenges can be classified into three main categories. First, there is insufficient size and variety of datasets because collecting images for rice leaves is very expensive and demands agricultural expertise for precise plant disease detection and identification^18–20^. Second, some rice leaf diseases have similar symptoms; even professionals or experts fail to identify them by eye. One symptom may vary because of geographic locations, weather conditions, and crop development^21^. Third, there are many problems in rice leaves images like noise, illumination, and low contrast^22^. There is a great advancements in image preprocessing and computer vision techniques in various fields which help in detection and classification rice plant diseases.

The motivation for this paper can be initiated from the existence of different categories of feature extraction methods, including methods that have not been utilized before in this research area, to the best of the author’s knowledge. Therefore, this paper aims to extract discriminative features using a multi-level handcrafted feature extraction technique to improve the classification accuracy of various rice plant diseases while addressing its related challenges. Therefore, the paper’s main contributions are summarized as follows:

- Handling dataset imbalance problem using data augmentation techniques.
- Enhancing the uncontrolled illumination conditions during image acquisition using the logarithmic transformation image preprocessing technique.
- Combining color features by Color Correlogram and Color Texture features using LBP provides complementary and discriminative information.
- It is the first attempt to apply the Multi-Level (ML) method for feature representation and the Multi-Channel Local Binary Pattern (MCLBP) method for extracting color and texture features from images for classifying rice plant diseases.
- The proposed system has been evaluated on three benchmark datasets to validate our results and achieve the generalization concept.
- Many comparisons between color and texture feature extraction techniques and classifiers have been implemented.
- Many comparisons according to average accuracy and processing time to ensure the reliability of this proposed system with the TensorFlow models.
- Using the one-against-all support vector machine (SVM) classifier, the proposed feature extraction methods outperform previous research attempts to classify rice plant diseases.

The paper’s remainder is structured as follows. "Related work" discusses the related research for rice plant leaf disease detection and classification. "Proposed framework" presents the proposed method of this paper for the classification of rice leaf diseases. "Experimental results" discusses the comparisons and experimental results. "Results and discussion" discusses the results and discussion. Finally, the conclusion and future directions are presented.

## Related work

Recently, different approaches have been proposed for plant disease detection and classification^23–26^. These approaches were evaluated on different datasets with various characteristics. From these datasets, we utilized three benchmark datasets^27–29^ for our evaluation. Then, to develop a plant disease detection system, the plant images must be effectively represented using discriminative features. The techniques used for feature extraction could be generally classified into two categories: handcrafted feature-based and deep learning-based techniques. The following subsections present the previous studies for plant disease detection classified according to feature extraction technique. Additionally, a comparison of these papers is discussed in Table 1.

### Handcrafted features-based studies

Handcrafted methods depend on different techniques for manual feature extraction^30^. These methods are used with feature vectors of different methods trained for classifying features related to each rice disease. After that, the ML classifier was then used to identify some new images of rice leaf diseases. The extracted features are color, shape, and texture. Below, we list some research attempts proposed for manual feature extractions.

Prajapati *et al.*^31^ discussed an approach for rice leaf disease detection and classification method. This paper worked with 2800 images from two datasets with five classes (BLB, TU, SB, BL, and BS). First, the background of the images was removed. After that, Kmeans clustering was applied to segment the disease part. Then, feature extraction was applied by extracting color ((R, G, B) mean, (L, A, B) mean, (H, S, V) mean, standard deviation, Kurtosis, Skewness), shape (minimum area and maximum area of diseased spots), and texture (contrast, energy, correlation, homogeneity, grayscale co-occurrence matrix (GLCM) properties) features. Finally, the SVM classifier was applied. This paper achieved 62% for accuracy.

Asfarian *et al.*^32^ discussed a method for classifying paddy diseases. This paper was applied to public datasets with three classes: BLB, TU, BL, and BS. This paper applied ’S’ component descriptors for every lesion image; the classification process was applied through the probabilistic neural networks. This paper achieved 83% for accuracy.

Pinki *et al.*^33^ proposed a method for the classification of paddy leaf diseases of public datasets with three classes (BLB, BL, and BS). First, image processing and contrast enhancement were applied to images. Then, color features like entropy, correlation, wavelet packet entropy, standard deviation, kurtosis, contrast, energy, variance, and root mean square (RMS). Finally, the SVM classifier was applied. This paper achieved 92.06% for accuracy.

Saha and Ahsan *et al.*^34^ proposed a method for classifying rice leaf diseases of two collected public datasets with five classes: BLB, TU, SB, BL, and BS. Statistical and textural features were extracted using the GLCM method. 64 texture features, like one-dimensional color information and six critical structural features, were extracted. Random forest (RF) was applied for the classification process with 92.77% for accuracy.

Chaudhary *et al.*^35^ proposed a method for rice plant disease detection and classification. This method used GLCM and Intensity-Level Based Multi-Fractal Dimension (ILMFD) for the features extraction step. Many classifiers have been applied, such as Support Vector Machine (SVM), Artificial Neural Network (ANN), and Neuro-Genetic Algorithm (Neuro-GA). SVM achieved an accuracy of 96.7%.

### Deep learning feature-based (DL) studies

Deep learning is a branch of ML applied using the neural network for feature learning. The deep learning concept is similar to how the human brain works. The feature extraction has been made through various hidden layers. Deep learning methods extract high-level features from rice plant leaf diseases that help in precise disease identification^36^.

Sethy *et al.*^37^ used the deep learning method to classify rice leaf diseases. This paper made many experiments using different methods such as handcrafted methods such as LBP, histogram of oriented gradients, GLCM method plus SVM classifier, and deep learning methods, such as convolutional neural networks (CNN) and ResNet50. ResNet50, then the SVM classifier achieved higher results than other methods. This method achieved an accuracy of 79.58 % for AlexNet and 98.38 % for ResNet50.

Ganesan and Chinnappan^38^ proposed a method for paddy leaf diseases. First, contrast enhancement and filtering were applied to the dataset with four disease classes (BLB, BL, BS, and TU). Second, adaptive K-means clustering was applied for the segmentation of the abnormal region of the paddy leaf. Finally, the recognition process was applied using the ResNet for feature extraction, and you only look once (YOLO) network for classification. It achieved 97.67% for accuracy.

Rahman^39^ depended on deep learning approaches in rice plant image disease detection and classification. This paper applied VGG16 and InceptionV3 and, after that, fine-tuned for rice disease detection and recognition. This code was applied to the public dataset that contained 1426 images with nine classes. The experimental results achieved 93.3% for accuracy. This paper suffered from a lack of images in the used dataset.

Ghosal and Sarkar^40^ presented a method for rice plant disease detection and classification using deep learning techniques. This paper created a private dataset of 2156 images with four classes: BL, BS, BLB, and healthy. This method depended on the VGG-16 pre-trained model and achieved 92.46% for accuracy. The limitation of this paper is the lack of images in the dataset that affected accuracy.

Wang *et al.*^41^ proposed the attention-based depthwise separable neural network with Bayesian optimization (ADSNN-BO) for rice plant disease detection and classification. This method was based on the augmented attention method and MobileNet structure. The experiments were applied on a public dataset with 2370 images of four classes (BS, BL, rice hispa, and healthy). This paper achieved 94.65% for accuracy.

Mohapatra *et al.*^42^ proposed a method that depended on deep learning for rice plant disease classification. This paper worked on a public dataset with four classes: BL, BLB, BS, and TU. This paper applied deep learning models such as DensNet121, VGG19, ResNet152, InceptionV3, and custom-CNN. This paper applied data augmentation on this dataset to solve the imbalance problem. The custom-CNN model achieved 97.47 % for accuracy.

Daniya and Srinivasan^43^ presented a rice plant disease detection and classification method. This paper used a public dataset with four classes (BLB, BL, BS, and TU). This paper applied shuffled shepherd social optimization-based deep learning (SSSO-based deep learning) for rice disease classification. This process was carried out using a deep maxout network and long short term memory (LSTM) model. This method achieved 92.6% for accuracy. Haruna *et al.*^44^ presented a method for rice plant leaf disease detection and classification. This paper worked on a dataset containing four rice disease types: TU, BLB, BL, and BS. It depended on a deep learning method based on style-generative adversarial network adaptive discriminator augmentation (SG2-ADA) and also used a Laplacian filter to enhance the system performance. It achieved 91.83% for accuracy.

0Dai *et al.*^45^ proposed a method for plant disease detection and classification based on a deep learning model (PPLC-Net). This model consisted of convolution, GAP layers, and a multi-level attention mechanism. It applied data augmentation to generate more images and applied robustness and generalization of feature extraction. It achieved 99.70% accuracy. Arya *et al.*^46^ presented a method for the detection and classification of rice plant diseases based on transfer learning. The combination between MobileNetV2 and Inception networks has been applied, and finally, a fully connected layer has been added for classification. The k-fold cross-validation was used to reduce the training bias and achieved 98.75%.

**Table 1:** A summary of previous studies for the detection and classification of rice plant leaf diseases using different feature extraction techniques.

Publication	FE/Classification	Detected Diseases	Dataset	Accuracy%	Strengths	Limitations
Prajapati *et al.* ^31^ (2017)	Features as color (mean of (RGB), (LAB), and (HSV), standard deviation, Kurtosis, Skewness), shape (Minimum and maximum area of diseased spots), and texture (GLCM properties). SVM for classification.	BLB, BL, and BS	120 images of rice leaf diseases (3 classes)	62	Fusion between color and texture features	Small number of images in dataset.
Asfarian *et al.* ^32^ (2013)	‘S’ component descriptors and probabilistic neural networks classifier	BLB, TU, BL, and BS	305 images of rice leaf diseases (4 classes)	83	Identify 4 types of rice diseases using fractal descriptors	Low contrast images and small number of images in dataset
Pinki *et al.* ^33^ (2017)	color features such as entropy, correlation, Standard deviation, Kurtosis, Contrast, Energy, Variance, Root Mean Square (RMS) and SVM classifier.	BLB, BL, and BS	120 images of rice leaf diseases (3 classes)	92.06	Kmeans segmentation code enhanced accuracy results	Illumination problems in images
Saha and Ahsan *et al.*^34^ (2021)	GLCM for feature extraction and Random forest.	BLB, TU, SB, BL, and BS	Two collected public datasets 2800 images of rice leaf diseases (5 classes)	92.77	Identify 5 classes of rice diseases	Low contrast images
Chaudhary *et al.* ^35^ (2024)	Features as GLCM and ILMFD and SVM classifier	BLB, BS and LS	4684 images with (3 classes)	96.7	Texture features	Low accuracy
Sethy *et al.* ^37^ (2020)	Transfer learning (AlexNet), ResNet50 with SVM classifier	BLB, BL, BS and TU	5932 images of diseased rice leaf (4 classes)	79.58, 98.38	Deep learning usage	illumination problems in images
Ganesan and Chinnappan ^38^ (2022)	Resnet for feature extraction and YOLO for classification	BLB, BL, BS and TU	5932 images of diseased rice leaf (4 classes)	97.67	Hybrid deep learning models usage	Low contrast and illumination problems
Rahman *et al.* ^39^ (2020)	VGG16 and InceptionV3	False Smut, BLB, neck blast, rot, SB, brown plant hopper, hispa, stemborer, and BS and others	1426 images of diseased rice leaf with (9 classes)	93.3	Identify 9 classes of rice diseases	Small number of images in dataset
Ghosal and Sarkar^40^ (2020)	VGG-16	BL, BLB, BS and healthy	2,156 images of diseased rice leaf with (4 classes)	92.46	Using CNN with transfer learning	Low contrast images
Wang *et al.* ^41^ (2021)	ADSNN-BO model	BS, BL, rice hispa, and healthy	2370 images of diseases rice leaf with (4 classes)	94.65	Using ADSNN-BO model with MobileNet	Illumination problems in images
Mohapatra *et al.*^42^ (2022)	Custom-CNN	BLB, BL, BS and TU	5932 images of diseased rice leaf (4 classes)	97.47	Using VGG16 model	Low contrast images
Daniya and Srinivasan ^43^ (2023)	SSSO-based deep learning	BLB, BL, BS and TU	5932 images of diseased rice leaf (4 classes)	92.6	Using optimization with deep learning	Illumination problems in images
Haruna *et al.* ^44^ (2023)	Style-Generative Adversarial Network Adaptive Discriminator Augmentation (SG2-ADA)	BLB, BL, BS and TU	5932 images of diseased rice leaf (4 classes)	91.83	Using GAN algorithms	Illumination problems in images
Dai *et al.* ^45^ (2023)	PPLC-Net	22 diseases as BL ... etc	4503 images (22 classes)	99.7	Highest accuracy	Weather data parameters
Arya *et al.* ^46^ (2024)	MobileNetV2 and Inception	BLB, BL, BS and TU	5932 images of diseased rice leaf (4 classes)	98.75	Transfer learning	No features selection used

### Studies limitations

As this section discusses many papers on detecting and classifying rice plant diseases, they still have problems such as:

- **Low detection accuracy:** One of the basic concerns through previous papers reviewing is the low detection accuracy of the models used. This is a big problem because precise detection is crucial for detecting and effectively classifying rice plant diseases. Low accuracy leads to the failure to detect rice diseases, resulting in serious consequences for food security and crop yields.
- **Dependency on only accuracy:** The dependency on only average accuracy as the sole metric in many studies represents a critical shortcoming. Average accuracy gives a global sense of the system’s performance but doesn’t explain how it performs through various dataset classes. For example, diseases that are harder to diagnose or less common might not be fully represented in the accuracy overall, which could lead to inaccurate findings. The full efficacy of these models is yet unknown because measurements like precision, recall, and F1-score for each disease class are missing. This is particularly problematic when it comes to differentiating between illnesses that have similar symptoms.
- **Rice images problems:** The image quality is very important and helps in rice plant disease detection and classification. Many studies struggle with various problems in image acquisition, such as noise, low contrast, and poor illumination caused by the image-capturing process. These problems greatly affect rice plant disease classification systems as they have variability in rice images that make the systems unable to detect the disease.
- **Similar disease symptoms:** Rice plant diseases often have similar symptoms that increase the complexity of the systems to detect and classify these diseases. This problem leads to misdiagnoses and decreases the system’s performance. This problem is a big challenge for machine learning and deep learning models that may struggle to differentiate between rice diseases.
- **Limited datasets:** The small rice image number is a critical problem that restricts the system’s ability to generalize and learn effectively. This problem means the system may perform well on specific datasets they learned and fail to test unseen or new data.

To overcome the aforementioned problems, a method for classifying rice plant diseases is proposed that helps in solving many problems. First, the data augmentation method is used to solve dataset imbalance and the small number of images. Second, the log transformation method solves the illumination problem. Third, many methods used depended on only one type of feature extraction from all images, resulting in low accuracy, so multi-level is used for feature representation followed by MCLBP, which helps increase method accuracy.

## Proposed framework

**Fig. 2:**
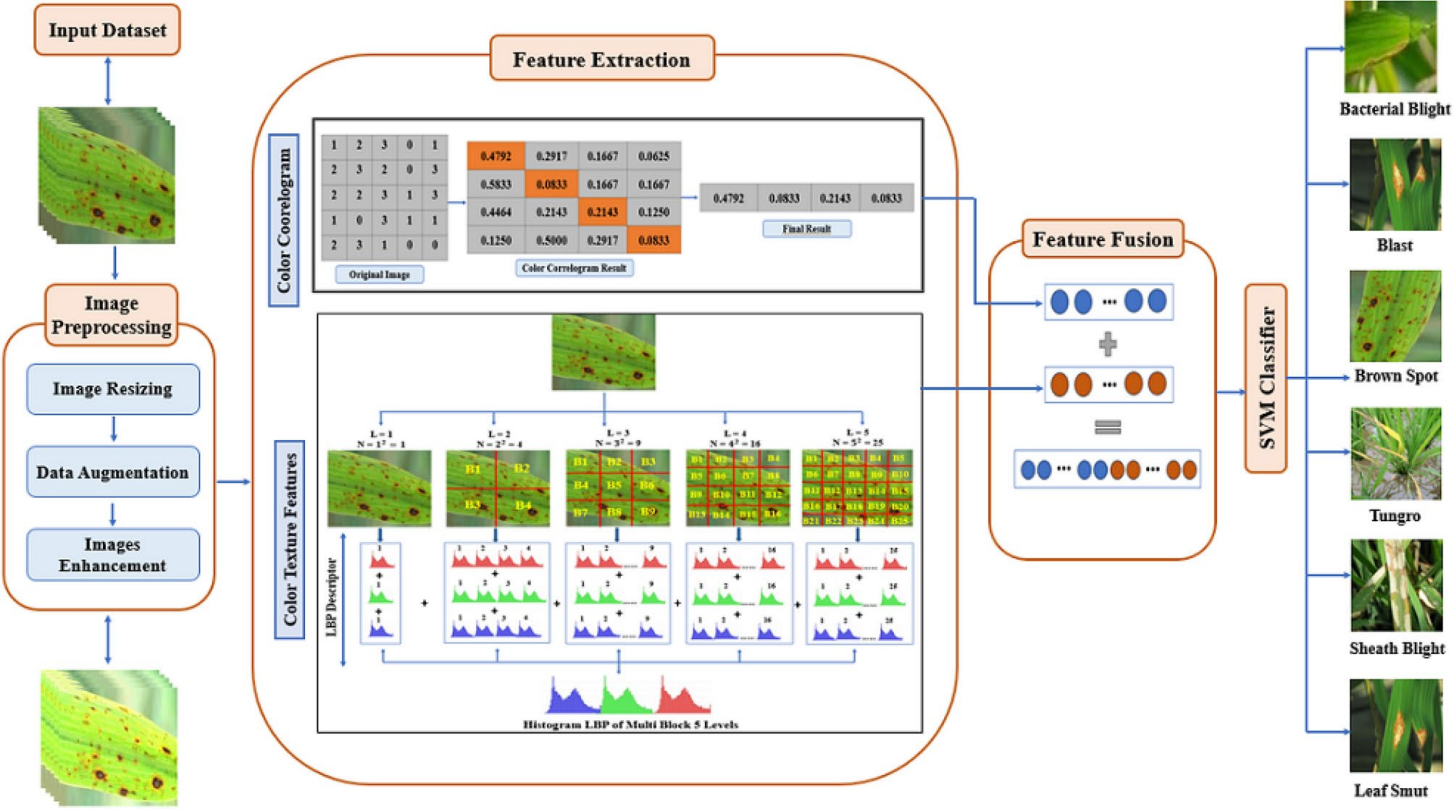
The framework of the proposed rice diseases classification system.

As mentioned, existing plant disease classification systems, specifically rice plants, suffer from low performance resulting from inefficient feature extraction techniques. Therefore, this paper presents a robust system for classifying rice plant leaf diseases using a novel multi-level-based feature extraction approach. Several rice plant diseases could be identified: BLB, BL, BS, TU, SB, and LS. The proposed system combines Five main stages, as shown in Fig. 2. The five stages of the proposed framework are image acquisition, preprocessing, features extraction, features fusion, and image classification. First, we obtain input images of rice plants from three benchmark datasets as^27–29^, the first dataset with four disease types as BLB, BL, BS, and TU, the second with five diseases as BLB, BL, BS, TU, and SB and the third dataset has three diseases as BLB, BS, and LS. Then, the obtained images are preprocessed using data augmentation followed by image enhancement techniques. Then, in the features extraction step, color features are extracted using a color correlogram, and the multi-level-based feature extraction approach extracts color texture features from the preprocessed images using LBP. After that, the features fusion step has been applied for combining the features extracted. Finally, the extracted features were fed into SVM for classification. A detailed discussion of these stages is presented in the following subsections. Figure 8 indicates the flowchart of the rice plant diseases classification system and also algorithm 1 presents the step of this proposed system.

### Dataset acquisition

Three benchmark datasets have been tested in this paper.

- **First Dataset (DS1): ** The first dataset was called Rice Leaf Disease Image Samples hosted by Mendeley Data. It was provided by the agricultural site of Sambalpur University and Bargarh district, Odisha, India. It was proposed by (Sethy et al., 2020)^27^, that was taken from farming areas in the Balasore District, which is a part of eastern Odisha, and some images of rice diseases were also retrieved from online sources. The dataset consisted of 5932 images of infected rice leaves with four types of diseases: BLB, BL, BS, and TU. Each class has been used to train and test the proposed methodology. This dataset is made available in the Mendeley archive data. Table 2 indicates the image count in each class.
- **Second Dataset (DS2):** The second dataset was called Rice Diseases, which was hosted by Kaggle. The images in the dataset were collected from several sources, real world, and automatic capturing. This dataset consists of five types of rice image diseases: BLB, SB, BS, BL, and TU. These images were collected from two different datasets^28,34^. The image number in this dataset is 2800 images, and each class contains 560 images. Table 2 indicates the image count in each class.
- **Third Dataset (DS3):** The third dataset is called Rice Leaf Diseases Dataset which was hosted by Mendeley Data. This dataset is a big collection of leaves images that focused on three major diseases such as BLB, BS, and LS that affect rice plants. Antony and Prasanth proposed this dataset^29^ to help machine learning practitioners, agronomists, and researchers analyze, diagnose, understand, and predict the rice plant disease occurrence based on many parameters and attributes. These images were high resolution, exhibiting many symptoms of the disease. This helps in the visual diagnosis and classification of rice diseases. The count of images in this dataset is 4684 for three classes. Table 2 indicates the image count in each class.

Fig. 1 presents the samples of two tested datasets. Table 2 indicates the description of this dataset. Cross-validation is an essential technique in statistical modeling and machine learning that helps evaluate the system performance by dividing data into several subsets. The K-fold cross-validation method is used for evaluation, where K refers to the number of groups or folds. 5 fold cross-validation method is applied in the proposed method. 5 fold cross-validation method is applied by dividing the dataset into five equal folds. Each fold has been used as a test set, and the other for the training model. Five iterations were applied to every dataset; all evaluation metrics were averaged. 5-fold cross-validation has many advantages over a single train-test split because various folds reduce overfitting risks and indicate how the model works on unseen data.

**Table 2:** The rice leaf disease dataset description.

**Datasets**	**Rice leaf disease**	**Number of Images**
DS1	BLB	1584
	BL	1440
	BS	1600
	TU	1308
	Total	5932
DS2	BLB	560
	BL	560
	BS	560
	TU	560
	SB	560
	Total	2800
DS3	BLB	1604
	BS	1620
	SL	1460
	Total	4684

Consider dataset which has been called *D*, containing images of rice plants referred to as *I*. It could be mathematically formulated as shown in Eq. 1.

1 $$\begin{aligned} D = \{I_1, I_2, I_3, ... I_K\} \end{aligned}$$

where k is the number of images in the dataset.

### Image preprocessing

Acquiring images of rice plants using a camera is affected by many factors. These factors include weather conditions, sun angle, and illumination, affecting image quality. Therefore, image preprocessing is required for obtaining an enhanced high-quality image^47,48^. The image preprocessing stage involves three main phases: image resizing, data augmentation, and image enhancement, as discussed below.

#### Image resizing

All input rice images are resized into $224 \times 224$ to unify the size of images in all classes of datasets used. Eq. 2 refers to the image resizing operation. An example of a resized rice image is shown in Fig 2.

2 $$\begin{aligned} D_{\text {resized}}(x, y) = D_t\left( \frac{x}{s}, \frac{y}{t}\right) \end{aligned}$$

where D_resized indicates the resized images in the dataset, s is the scaling factor along the x-axis, and t is the scaling factor along the y-axis.

#### Data augmentation

**Fig. 3:**
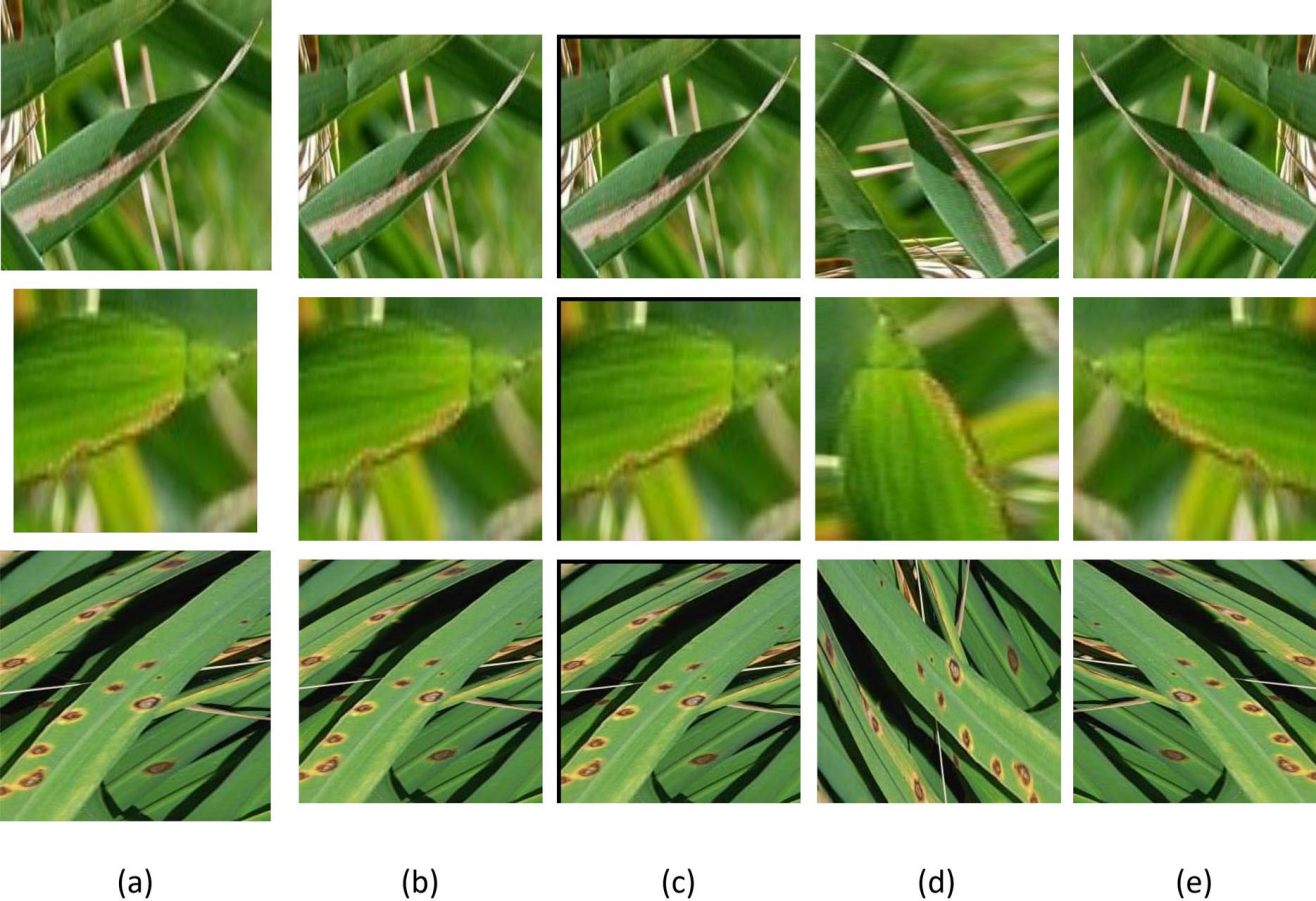
The result of transformation operations on three image samples from the dataset: (**a**) Original images, (**b**) Image resizing, (**c**) Translation operation, (**d**) Rotation operation, and (**e**) Flipping operation.

Data augmentation is applied to create more images in the dataset size and solve the problems of overfitting^49^, which helps solve the dataset imbalance problem, resulting in an unequal number in each class. Data augmentation is applying simple changes to the original images to generate new images. These operations are translation, flipping, and rotation performed on original images^50^. The translation is applied by shifting the image across the x and y-axis. The images are translated between $-5$ to $+15$. The flipping operation makes a mirror image of the input image on the opposite line side. Rotation is used to rotate the images by $-10$ to $+10$ degrees. Some dataset images have been chosen randomly, and some transformation operations have been applied. Fig. 3 presents some operations on images from the tested dataset. These operations are mathematically formulated in the following equations.

3 $$\begin{aligned} D_t = T(D) \end{aligned}$$

where D_t is the dataset after applying some transformations on images, T refers to the applied transformation operations: translation, flipping, and rotation as defined in Eq. 4, Eq.5, and Eq. 6 respectively.

4 $$\begin{aligned} D_{\text {translated}}(x, y) = D_t(x - \Delta x, y - \Delta y) \end{aligned}$$

where D_translated(x, y) denotes the translated images in dataset, $\Delta x$ is the amount of translation in the x-axis and $\Delta y$ is the amount of translation in the y-axis.

5 $$\begin{aligned} \begin{aligned} D_{\text {horizontalFlipped}}(x, y) = D_t(width - x -1, y) \\ D_{\text {verticalFlipped}}(x, y) = D_t(x, height - y - 1) \end{aligned} \end{aligned}$$

where D_horizontalFlipped(x, y) indicates the horizontal flipped images in the dataset, D_verticalFlipped(x, y) indicates the vertical flipped images in the dataset, width indicates image width, and height indicates image height.

6 $$\begin{aligned} D_{\text {rotated}}(x, y) = D_t\left( x \cdot \cos (\theta ) - y \cdot \sin (\theta ), x \cdot \sin (\theta ) + y \cdot \cos (\theta )\right) \end{aligned}$$

where D_rotated(x, y) indicates the rotated images in dataset, $\theta$ is the rotation angle in degrees, and $\cos (\theta )$ and $\sin (\theta )$ are the trigonometric functions cosine and sine, respectively.

#### Rice image enhancement

Image enhancement is applied to enhance the visibility and quality of images. Many problems affect images of rice plant leaves, such as noise, weather conditions, and illumination. This paper addresses the illumination problem, so the logarithmic transformation method is applied to solve this problem.

**Fig. 4:**
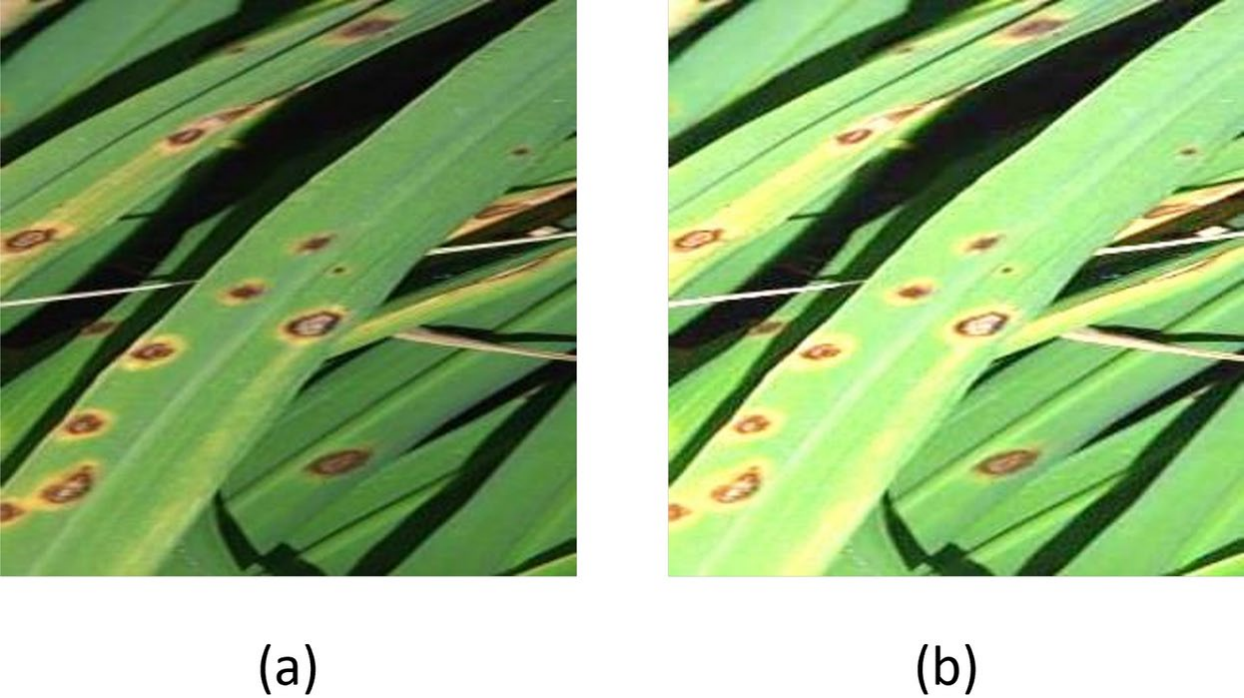
Enhancement process using log transformation: (**a**) Original image and (**b**) Enhanced image.

**Logarithmic (Log) transformation** is one of the basic image enhancement techniques that is applied to enhance the image contrast^51^. This transformation maps the low, narrow-range images into a wider range of output levels. After applying the logarithmic transformation, the darker intensities become brighter, making details present in images more visible to human eyes. Fig. 4 presents the enhancement process. First, the normalization process is applied to dataset images to achieve narrow-range pixels in images as in Eq. 3.2. Normalization is dividing each of its pixel values by the maximum value, which is 255. After that, the Log transformation method is applied as in Eq. 8.

7 $$\begin{aligned} \begin{aligned} D_{\text {tNorm}} = D_t/255 \end{aligned} \end{aligned}$$

where D_tNorm is the dataset after applying normalization

8 $$\begin{aligned} \begin{aligned} D_{\text {tLog}} = c*\log (1+D_{\text {tNorm}}) \end{aligned} \end{aligned}$$

where D_tLog is the dataset after applying log transformation for image enhancement, and c is a scaling constant. Its value differs according to the application used^52^, in this case the c value is set to 2 as in^53^.

### Feature extraction

Feature extraction is applied to recognize the intrinsic characteristics (i.e., features) of objects found in images. These extracted features are essential for describing the main and important information in mathematical form and classifying the classes. Color, texture, and shape features are extracted to classify rice plant leaf diseases. These features are used to distinguish between disease types of rice.

Shape features^54^ consists of area, minor/major axis length, perimeter, eccentricity, etc. Color features are based on various values used to identify the disease region^55^. Image texture is surface characterization for an object present in the image^56^. There are many techniques used for extracting texture features^57–59^, such as Gabor texture features^60^, GLCM^61^, gray-level run-length method (GLRLM)^62^, and LBP^63^.

In this paper, Color features have been extracted using color correlogram technique and color texture-based features are extracted using LBP. However, the novelty of this paper is applying LBP on multi-blocks of the image instead of the entire image using a multilevel feature representation approach^64,65^. These multi-blocks are used to increase image features, which in turn help classify rice plant image diseases efficiently. A detailed discussion of multi-block image representation is presented in the following subsection.

#### Color correlogram

Color Correlogram technique represents the spatial correlation of color changes with regard to the distance change in contrast to the color histogram that extracts only the color representation in an image and does not include any spatial information^66^. Color correlogram represents the distribution of color correlation of an image. It is used to retrieve content-based images. Firstly, the histogram was applied to an image subdivided into four equal bins, and every bin was subdivided into four bins with maximum frequencies. This information was stored in the correlogram form.

Color correlogram (CC) In general, CC shows how distance affects the spatial correlation of color pairings. An image’s CC is a table that is indexed by color pairs where the D entry for row ($c_{ii}$, $c_{jj}$ sets the probability to find a pixel of color $c_{jj}$ at a distance D from a pixel of color $c_{ii}$ in this image. CC is calculated as presented in Eq. 9

9 $$\begin{aligned} \gamma ^{(D)}_{c_{ii},c_{jj}}=Pr_{p_1\in I_{c(ii)},p_2\in I}[p_2 \in I_{c(jj)}\mid \mid p_1-p_2\mid =D] \end{aligned}$$

where $I$ indicates the image pixels set while $I_{c(ii)}$ is the pixels set with color $c_{ii}$, the colors in $I$ are quantized into $m$ colors $c_1,..., c_m$. $\gamma ^{(D)}_{c_i,c_j}$ is the probability for a given pixel $p_1$ with color level $ci$, another pixel $p_2$ is located at a distance $D$ in specific direction from the given pixel $p_1$ has a color level $c_{jj}$.

#### Multilevel (ML) representation

**Fig. 5:**
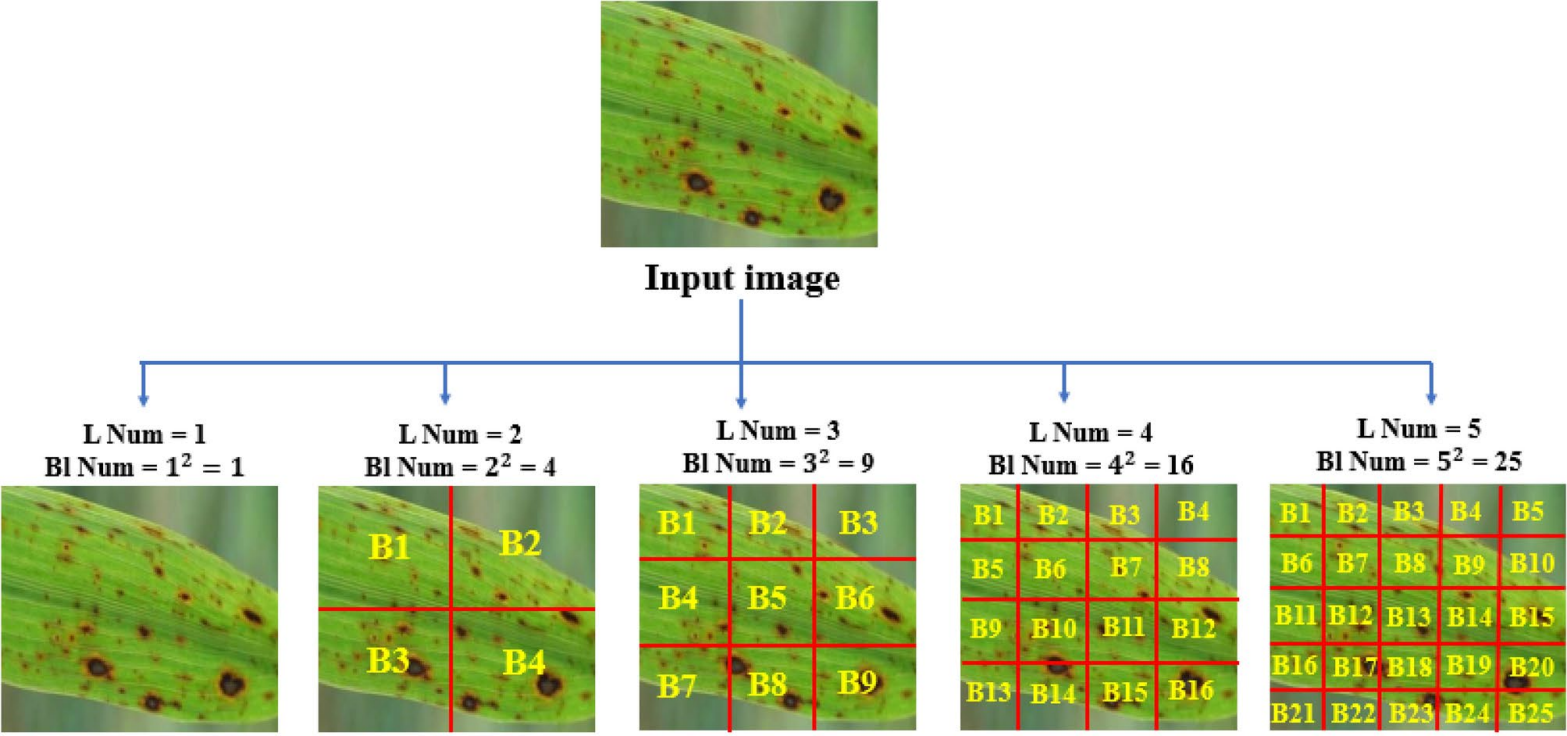
Multilevel feature representation into five levels, L represents the level number, and N represents block number.

The most common method for representing features is multilevel multi-block as a regular grid of fixed size^64,65^. ML method divides the input image into v$^2$ equal blocks where v is the number of levels for ML. The representation levels *v* is constructed from level 1, 2, 3, ... v ML representations. The process of ML representation can be mathematically formulated as illustrated in Eq. 10. Consider *L* is a positive integer that represents the level number such that $L>0$,

10 $$\begin{aligned} \begin{aligned} N = L^2 \\ D_{\text {tLog}} = \bigcup _{n=1}^N B_n \end{aligned} \end{aligned}$$

where N represents the number of blocks at level L, and B_n represents the nth blocks at specific level L. Fig. 5 illustrates the basic idea of the multilevel feature representation method. ML could be repeatedly applied with various levels *L* and corresponding blocks to extract color and texture features using LBP until reaching the best number of levels with the highest accuracy.

#### Color and texture extraction using LBP

Color and texture features provide essential information that helps detect and classify rice plant diseases. Texture is an important image investigation method for feature extraction and content analysis. It is used to describe the spatial arrangement between rice image pixels. Texture features are used in many applications as remote sensing imagery^67^, object recognition^68^, medical image analysis^69^, content-based image retrieval^70^, and object classification^71^.

Color and texture features provide essential information that helps detect and classify rice plant diseases. Texture is an important image investigation method for feature extraction and content analysis. It is used to describe the spatial arrangement between rice image pixels. Texture features are used in many applications as remote sensing imagery^67^, object recognition^68^, medical image analysis^69^, content-based image retrieval^70^, and object classification^71^.

The LBP method proposed by Ojala et al.^72^ is a vital texture descriptor in many computer vision applications. It extracts local texture features that are resistant to changes in illumination. This method is very fast and provides strong and significant discriminative power compared to other local texture feature descriptors. The LBP method is applied in many applications of texture classification^73–76^. LBP has many advantages, such as invariance to illumination, low computational complexity, implementation ease, and grey-scale variations robustness^77,78^.

**Fig. 6:**
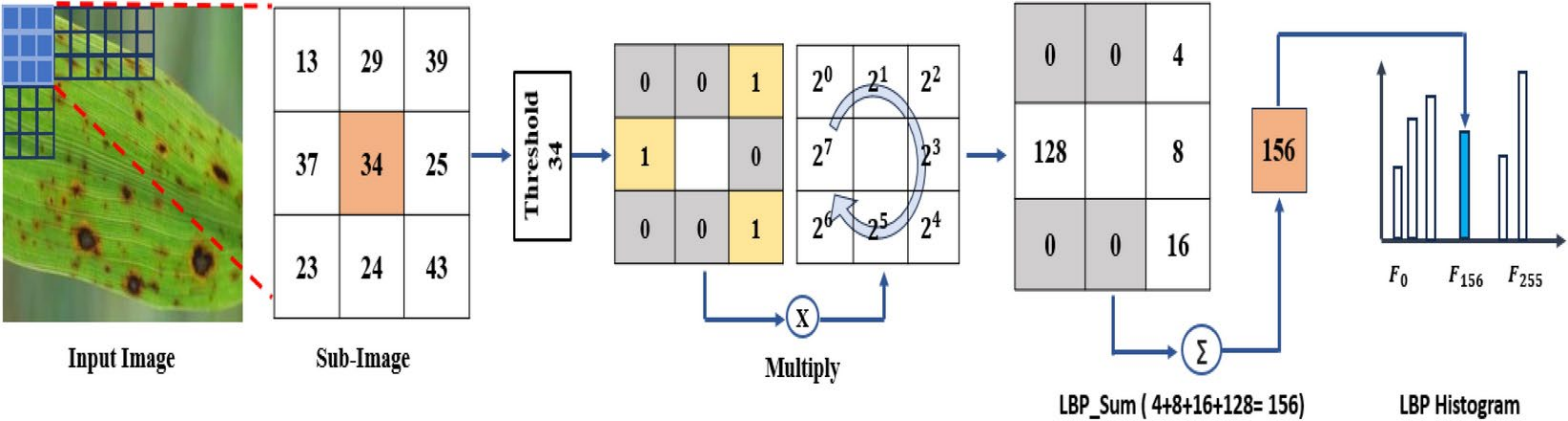
An example of LBP process on rice image for sub-image having neighboring pixels = 8, radius = 1.

LBP works as follows: first, it labels every pixel in the rice image by comparing the grey level with the neighboring pixels and giving a binary number. The value of unity is given to the neighbors with a grey level larger than the value of the center pixel in a predefined patch; otherwise, a zero value is given. The binary number is given to the center pixel. The normal LBP considers a 3 x 3 patch, so the pixels surrounding a binary number of 8 digits. Finally, all pixels are labeled, and the LBP feature map and histogram with 256 bins are generated. This LBP histogram is applied as a feature vector for the classification process, as each bin is considered one feature. Fig. 6 illustrates an example of the process of the LBP feature extraction defined in Eq. 11.

11 $$\begin{aligned} \begin{aligned} LBP(x, y) = \sum _{p=0}^N 2^p(g(B_p - B(x_c, y_c))) \end{aligned} \end{aligned}$$

where LBP(xc, yc) represents the LBP features at the xc, yc center pixel. B(xc, yc) is the value of the center pixel, and $B_p$ is the value of the neighbor pixel. The index p refers to the neighbor pixels index. If x is less than 0, the function g(x) will equal zero, otherwise it will equal one.

**Fig. 7:**
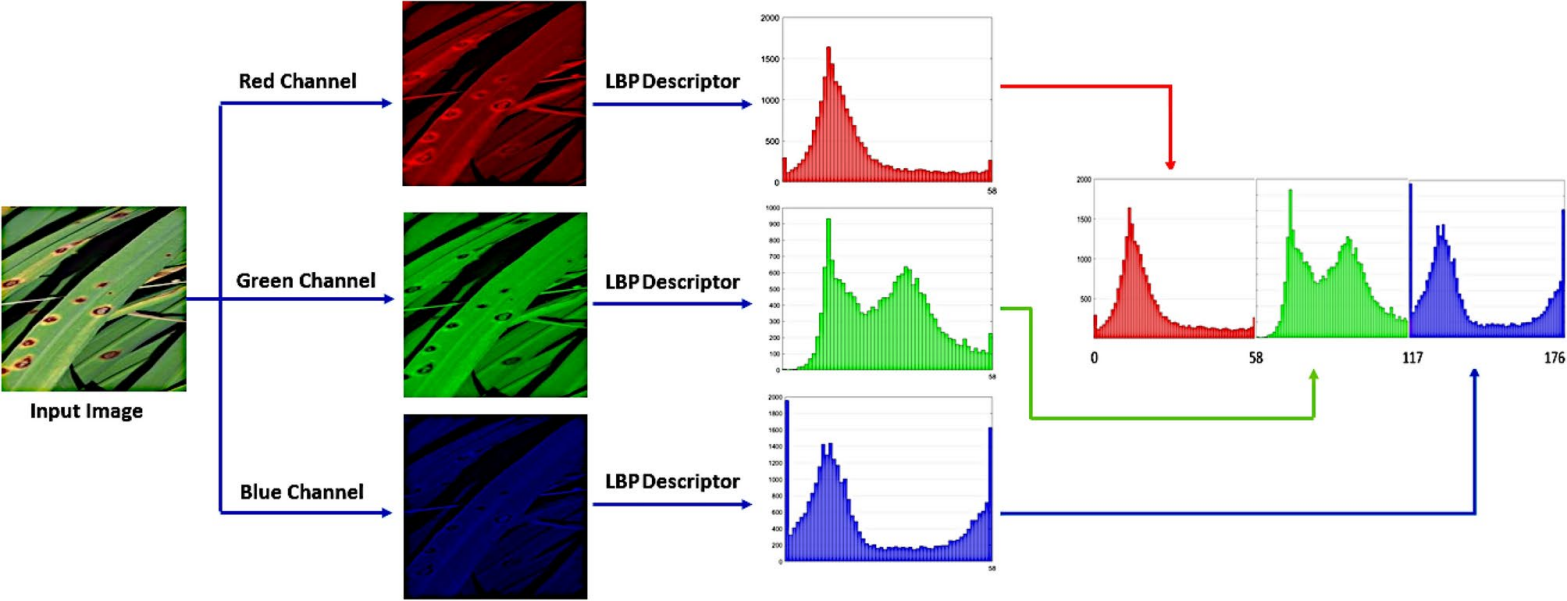
Extraction of RGB feature vector from a rice image using LBP.

Most papers work on classical LBP descriptors, and their variants have been used for gray-scale image preprocessing. The need for colored rice images over the internet, which is used in many applications, increases. Therefore, LBP descriptors have been utilized for extracting color-texture features from colored rice images. This is achieved by extending the LBP to process each color channel in the RGB-colored rice image as a simple gray-scale image^79^ as defined in Eq. 12 and illustrated in Fig. 7.

12 $$\begin{aligned} \begin{aligned} LBP(RGB) = LBP(Red)+ LBP(Green)+ LBP(Blue) \end{aligned} \end{aligned}$$

where LBP(RGB) represents the features extracted from red, green, and blue channels, LBP(Red) is LBP features from the red channel, LBP(Green) is LBP features from the green channel and LBP features from the blue channel.

### Features fusion

Feature fusion is an important technique in machine learning and data preprocessing that combines various features to generate more important, comprehensive, and robust data representation. It combines the complementary strengths of each feature extraction technique, improves the ability to have relationships and patterns through the data, and gives reliable and accurate results. Color features and color texture using a multi-level image representation based on the Local Binary Patterns (LBP) technique have been combined using the concatenation method as shown in Eq. 13.

13 $$\begin{aligned} FE = [\gamma ^{(D)}_{c_{ii},c_{jj}} + LBP(RGB)] \end{aligned}$$

where FE is the combined features between LBP(RGB) represents the features extracted from red, green, and blue channels and $\gamma ^{(D)}_{c_i,c_j}$ indicates the color features extracted using color correlogram.

### Rice disease classification

The classification is applied to distribute rice plant images into some classes and categories. The classification process is applied in two steps. First is the training process in which the classifier is created by applying the classification algorithms to learn the data and describing a predefined set of classes. Second is the testing process in which a subset of data is used to test the trained classifier model. SVM is applied for the classification stage.

SVM is one of the most widely applied machine learning for the classification process^80^. SVM algorithm in which the number of object data or features extracted are plotted as points in n-dimensional coordinate space. The corresponding features represent each coordinate. All points represent extracted features of all classes. After that, the hyperplane is found to differentiate between classes. Training vectors are used to find the hyperplane with maximum separation between classes. SVM kernels, such as linear, RBF, and polynomial, are tested, and linear has the best results. Linear SVM in this paper is used for multi-level SVM, especially one-against-all strategy^81,82^.

For the multiclass problem, SVM developed into multi-level SVM, especially a one-against-all strategy. It evaluates all classifier pairs by inducing a K binary classifier, where K is the number of classes.

The One-Against-All (OAA) method is often applied for extending the algorithms of binary classification, such as Support Vector Machines (SVM), to handle multiclass classification problems. In this method, if there are K classes, K separate binary SVM classifiers have been trained. Every classifier m has been trained to differentiate class m from all other classes combined. The decision method of every classifier depends on a linear kernel, and a hyperplane has linearly separated data. The objective is to find the optimal hyperplane defined by the weight vector $w_m$ and also bias term $b_m$ for every class m by minimizing the objective function as in Eq. 14

14 $$\begin{aligned} \begin{aligned} \min _{w_m, b_m, \xi _i}&\quad \frac{1}{2} \Vert w_m\Vert ^2 + C \sum _{i=1}^l \xi _i^m \\ \text {Constraints to}&\quad (w_m)^T FE_i + b_m \ge 1 - \xi _i^m \quad \text {for } y_i = m, \\&\quad (w_m)^T FE_i + b_m \le -1 + \xi _i^m \quad \text {for } y_i \ne m, \\&\quad \xi _i^m \ge 0, \quad i = 1, 2, \ldots , l. \end{aligned} \end{aligned}$$

where $FE_i$ is the extracted features for the i-th data point.$y_i$ represents the target labels of each data. $w_m$ is a weight vector related to class m. $b_m$ is a bias term associated with class m. $\xi _i^m$ are the slack variables allowing for soft margin classification. C is the regularization parameter used in balancing the classification error and margin size.

The kernel function that has been used in SVMs is:

15 $$\begin{aligned} K(x_i, x_j) = \langle \varphi (x_i), \varphi (x_j) \rangle \end{aligned}$$

This function calculates the inner product between the feature mappings $\varphi (x_i)$ and $\varphi (x_j)$ in a higher-dimensional space. This makes SVM to work in this higher-dimensional space efficiently.

In case of linear kernel, the kernel function is:

16 $$\begin{aligned} K(x_i, x) = x_i^T x \end{aligned}$$

Here, $K(x_i, x)$ indicates the dot product between the input vectors $x_i$ and $x$ in the original feature space. This kernel function has been used as the data is linearly separable.

**Fig. 8:**
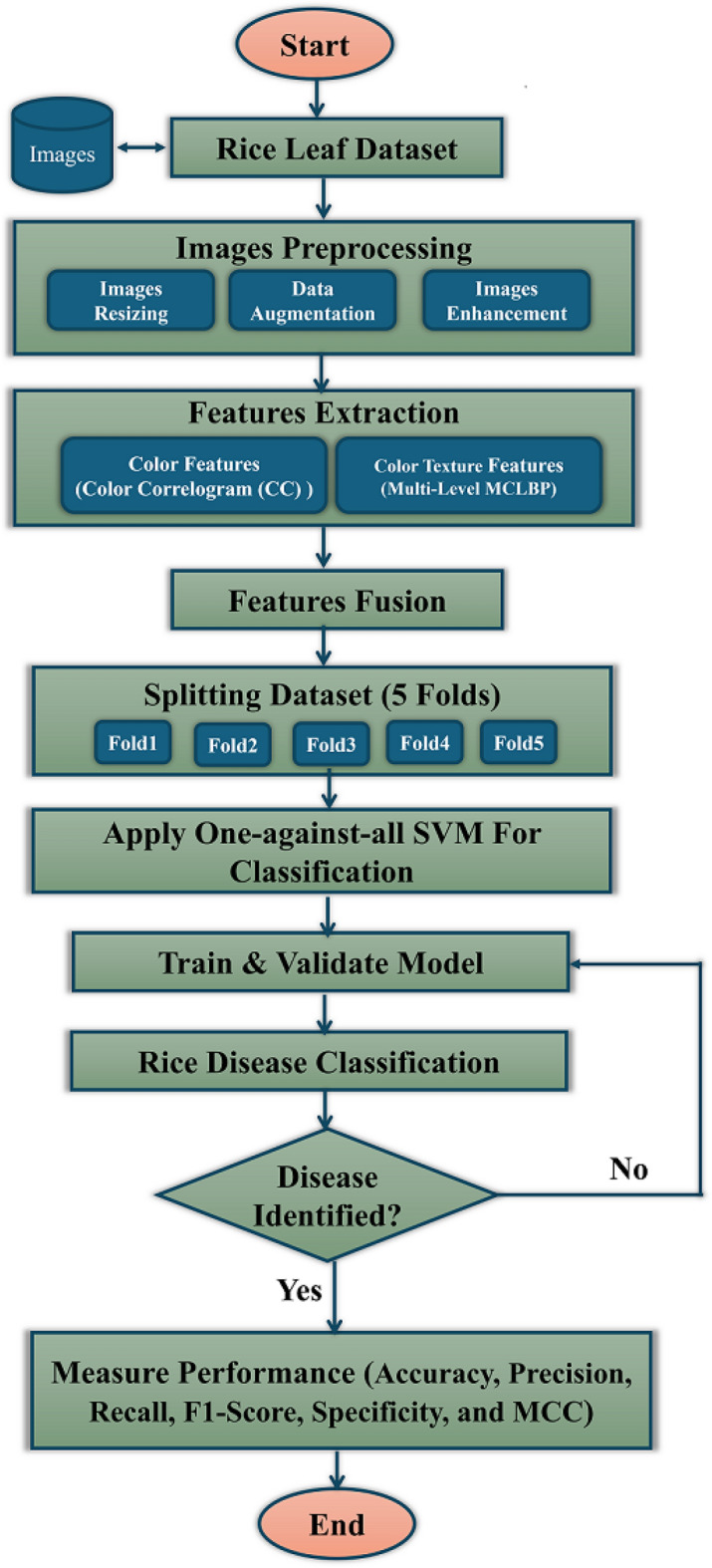
Flowchart of the proposed Rice leaf diseases classification system.

**Algorithm 1:**
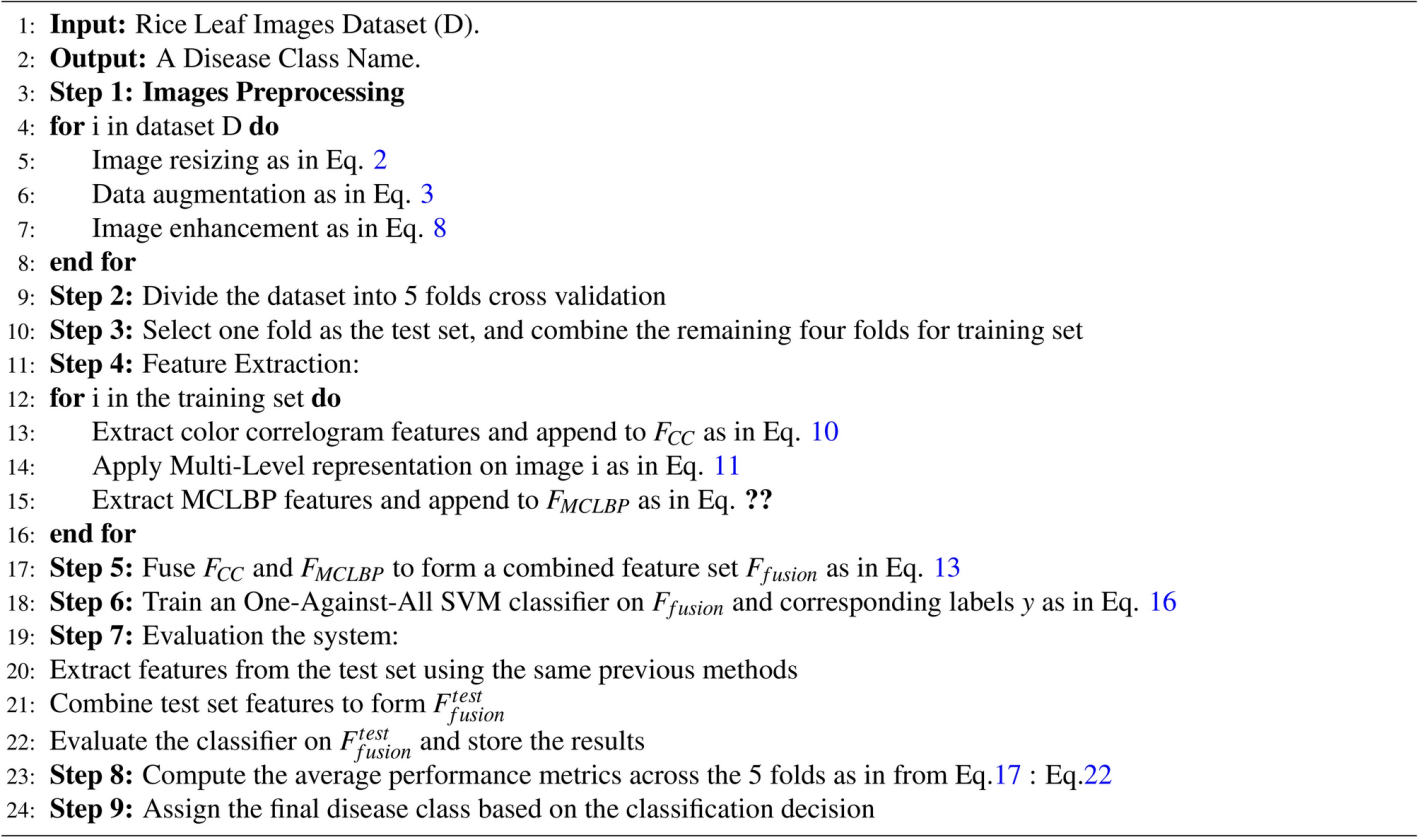
Rice leaf diseases classification.

## Experimental results

The proposed scheme’s performance for detecting rice plant diseases and classification is evaluated using a MATLAB computer simulation program (R2022b, MathWorks, Natick, MA, USA). This program is used for validating and testing several experiments. The computer configuration used in these experiments is described as follows: CPU: Intel(R) core (TM) i7-9750H CPU @2.60 GHZ (Lenovo, Beijing, China); Memory size: 16 GB RAM; OS: Microsoft Windows 10 (Microsoft, Redmond, WA, USA); the Coding: MATLAB R2018a. In the coming sections, we present the performance evaluation metrics.

### Performance evaluation metrics

There are multiple performance metrics used to compute the performance of the proposed method. This section discusses the mathematical formulations for the computation of these evaluation metrics. Some metrics are used for this evaluation, such as accuracy, precision, recall, specificity, F1-score and Matthews Correlation Coefficient (MCC). These metrics are computed by true positive (TP), false positive (FP), true negative (FN), and false negative (FN). TP is the number of cases correctly classified as belonging to a specific class. FP is the number of cases incorrectly classified as belonging to a specific class when they do not belong to that class. TN is the number of cases correctly classified as not belonging to a specific class. FN is the number of cases incorrectly classified as not belonging to a specific class when they belong to that class.

- **Accuracy:** It is computed for performance model measure which refers to the correctness of the system^83,84^. It is computed using Equation 17.

17 $$\begin{aligned} Accuracy = \frac{TP+TN}{TP+FN+FP+TN} \end{aligned}$$

- **Precision:** It is computed as the percentage of the number of correctly identified rice leaf classes with the disease (TP) to the overall number of leaf classes with the disease ((TP) + (FP))^85^. It is computed by Eq. 18

18 $$\begin{aligned} Precision = \frac{TP}{TP+FP} \end{aligned}$$

- **Sensitivity (Recall):** It is computed as the percentage of the number of correctly identified rice leaf classes with the disease (TP) to the overall number of leaf classes with the disease ((TP) + (FN))^86^. It is computed by Eq. 19.

19 $$\begin{aligned} Sensitivity = \frac{TP}{TP+FN} \end{aligned}$$

- **Dice Similarity Coeffient (F1-Score):** It is computed to estimate system quality^87^. It represents the balance between recall and precision. This measure is used when there is an imbalance in class distribution in data. It is computed by Eq. 20.

20 $$\begin{aligned} F1-Score = \frac{2*TP}{2*TP+FN+FP} \end{aligned}$$

- **Specificity :** It is the percentage of correctly recognized true negatives^88^. It means that some real negative data may in fact be projected as positive data and this process referred to as false positives. It is computed by Eq. 21.

21 $$\begin{aligned} Specificity = \frac{TP}{TP+FP} \end{aligned}$$

- **Matthews Correlation Coefficient (MCC) :** MCC is a measure that displays the correlation between observation and prediction^89^ by accounting for true and false positives as well as negatives, and providing a value between $-1$ and 1.

22 $$\begin{aligned} \text {MCC} = \frac{TP \cdot TN - FP \cdot FN}{\sqrt{(TP + FP)(TP + FN)(TN + FP)(TN + FN)}} \end{aligned}$$

## Results and discussion

This section presents the experimental results and comparisons conducted on the two datasets used in this paper. Some comparisons between texture and color feature extraction methods are presented. Also, comparisons between machine and deep learning classifiers have been conducted. Final subsection presents comparisons with state-of-the-art methods.

### A comparison of different feature extraction methods

Texture and color feature extraction techniques have important roles in detecting rice plant leaf disease and classification because they capture important information for visual characteristics of diseased plants. Combining texture and color features can achieve higher robustness and accuracy as it extracts chromatic and structural information in images.

Texture features are essential for rice disease detection and classification. Texture features are important in detecting and classifying rice plant leaf diseases as they extract unique and distinct variations and patterns on the rice plant surface. These texture methods analyze the pixel intensity distribution and capture important information. Therefore, many feature texture extraction methods are conducted to extract texture features, such as Weber local descriptor (WLD)^90,91^ which captures texture information in a manner that is resistant to changes in light and contrast by encoding the gradient orientation and differential excitation, GLCM^92,93^ computes the spatial relationship between pixels pairs at particular orientations and distances, local phase quantization (LPQ)^94,95^ extracts phase information from pixel neighborhood and it is very robust to blur variations, histogram of oriented gradients (HOG)^96^ which extracts the pixels gradients and generates the histogram of the orientations for these pixels, local frequency descriptor (LFD)^97^ concentrates on computing pixels intensities frequency, and LBP^78^ converts every pixel’s neighborhood into a binary pattern.

Color features are significant for detecting various changes in the rice leaves pigmentation that is a common symptom in multiple rice diseases. These features help differentiate between healthy and diseased rice leaves through color distribution analysis in leaves. There are many techniques for color feature extraction, such as Color Histograms extracting color content from an image^98^. This technique represents the color distribution by plotting every color’s frequency in an image. Color Moments extracts detailed statistical representation of color information through computation of the variance, mean, and skewness for each color channel as red, green, and blue in an image^99^, and Color Correlograms work on the idea of color histograms through spatial information incorporation, which represents how the colors of the pixels relate to pixels neighbors^66^. This technique extracts the image’s color frequency and spatial correlation.

We have three scenarios for applying these feature extraction methods. The first scenario applied FE methods on the entire image, the second applied FE methods on image blocks instead of the full image, and the third scenario applied fusion between multi-level color texture features and color features. We computed the predefined evaluation metrics for each scenario: precision, recall, F1-score, specificity, Matthew’s correlation coefficient (MCC), and accuracy.

#### Using the DS1

**Fig. 9:**
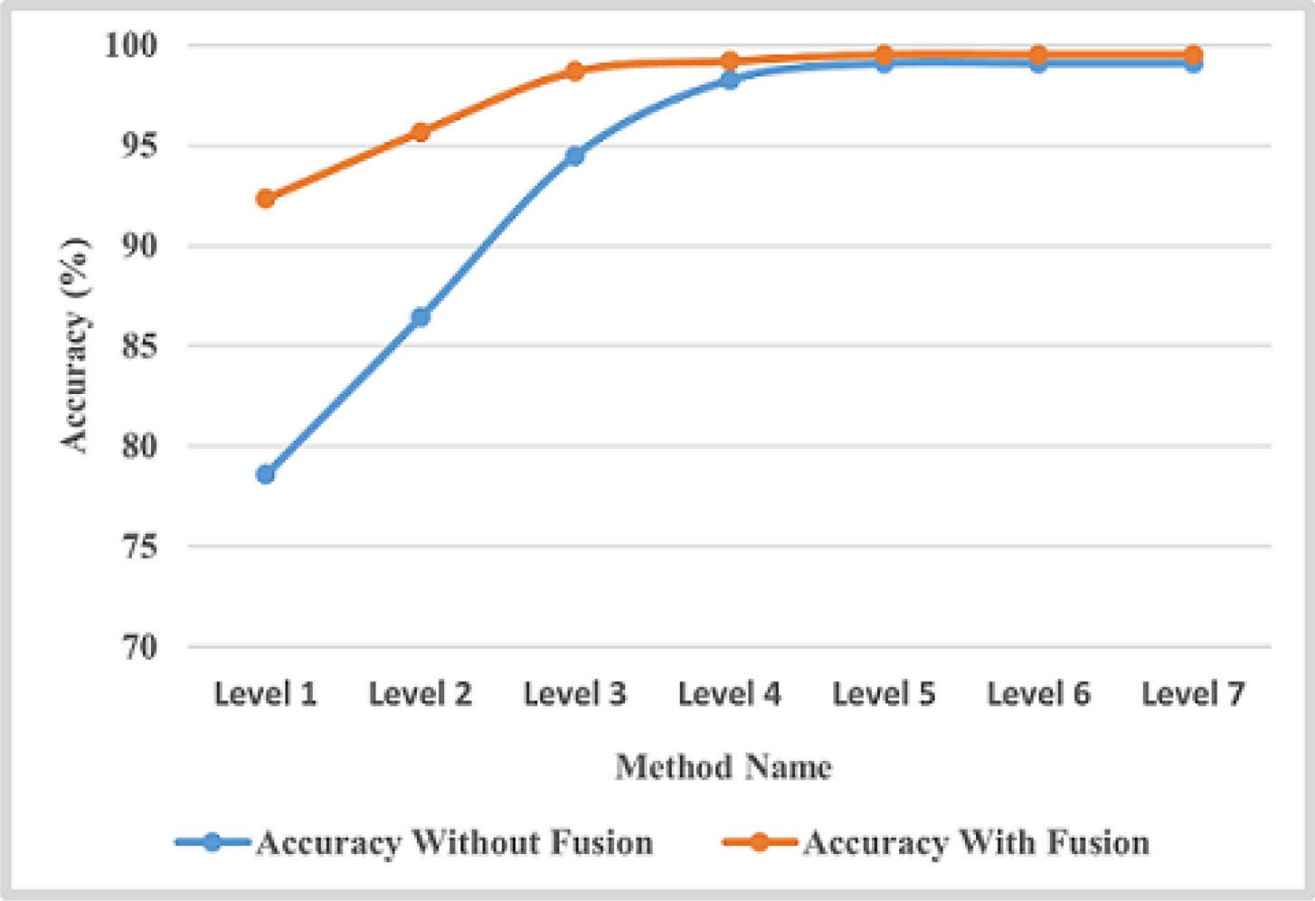
Overall accuracy of each level for the first dataset in case of without and with features fusion.

**Fig. 10:**
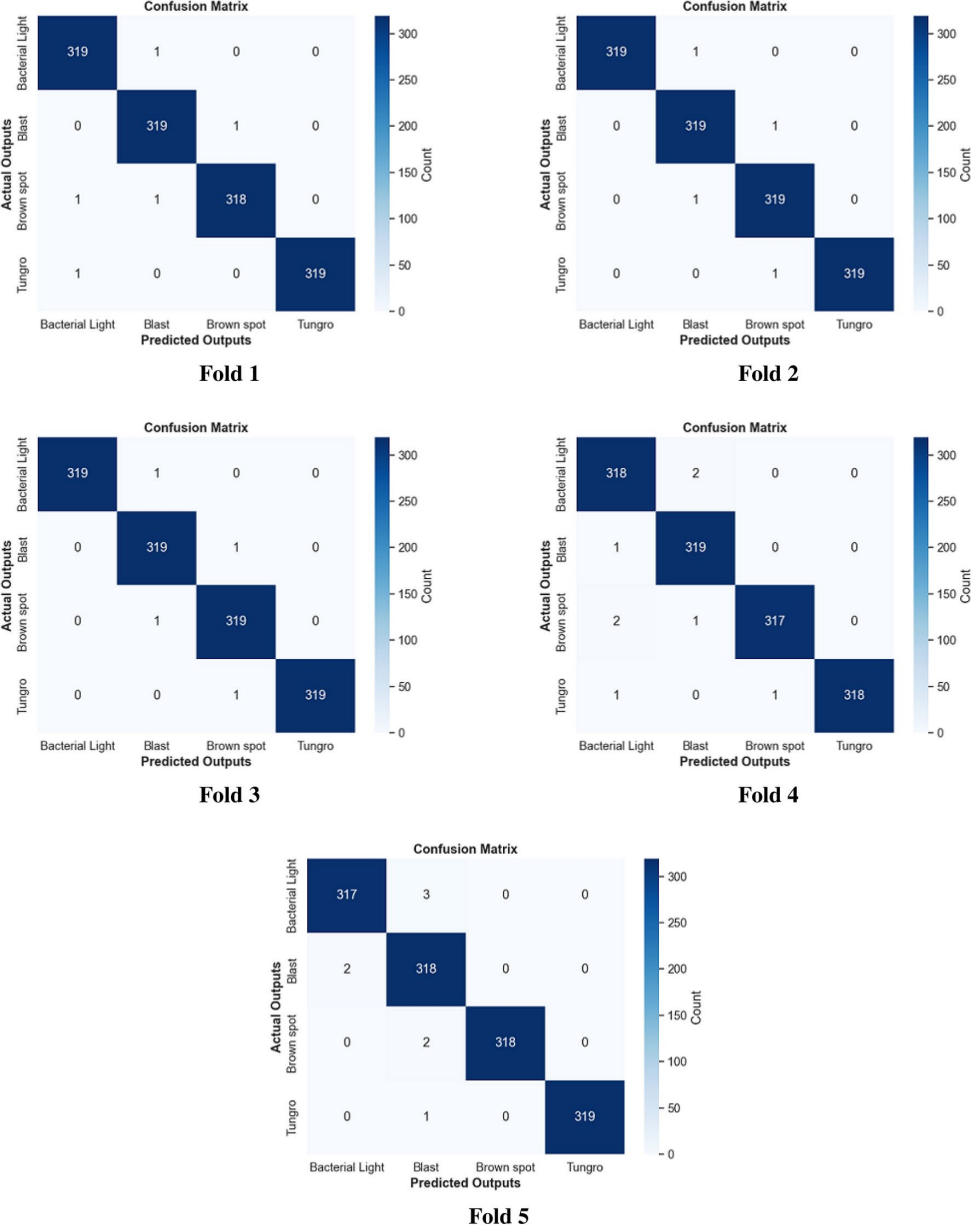
MCLBP ML (5 Levels) confusion matrix for 5 folds on the first dataset.

1. **The First scenario: Using the conventional full image representation**
  Different texture feature extraction techniques have been applied on the full image, and the results are shown in Table 3. Moreover, the overall accuracy results for these methods are shown in the last column in Table 3. From the results shown in Table 3, we noticed that the MCLBP method, a modified model of LBP that extracts features from each color channel in the image, outperforms the other feature extraction method. MCLBP achieved an overall accuracy of 78.59%, followed by LBP with 73.83%. Therefore, these methods (i.e., MCLBP and LBP) were utilized as feature extraction methods in the remaining experiments.
  According to color features extraction techniques, multiple techniques have been applied to the full image, such as color histogram, block color moment, and color correlogram. The experimental results have been shown in Table 4. The overall accuracy results for these methods are shown in the last column in Table 4. color correlogram color features extraction achieves highest results with 87.15% than other techniques.
2. **The second scenario: Using multi-level image representation**
  In this scenario, the feature representation method called multi-level (ML) has been applied, which divides the image into multi-blocks before extracting features. For ML representation, a relation exists between the level number and the number of blocks within it, as presented by Eq. 9.
  Regarding the number of levels used for representation, we tried different numbers until we reached stability. We started from level 1, and the stability begins from level 5 and converges, as shown in Fig. 9. Therefore, we used seven levels for representation. As noticed, multi-level representation of features has a significant effect on results. The more levels, the more accuracy. Then, for each level, we applied the LBP and MCLBP methods for feature extraction.
  **Using LBP:** The precision, recall, F1-score, and accuracy results are shown in Table 5. In addition, the overall accuracy of each level is shown in the last column in Table 5.
  **Using MCLBP:**, to enhance the results, multi-channel LBP (MCLBP) is applied instead of applying LBP. Table 5 presents the experimental results of precision, recall, F1-score, specificity, MCC, and accuracy. In addition, the last column in Table 6 presents the overall accuracy with respect to the number of levels. The results are stable from 5 levels and converge for higher levels, as shown in Fig. 9. Therefore, five levels are selected for computation.
3. **The Third scenario: Using fusion between color features and color texture with multi-level image representation**
  The fusion method between color features and color texture features with multi-level image representation is an important technique in image analysis, particularly in various tasks such as object detection and classification. Color features have been extracted using a color correlogram, which describes the distribution of colors inside an image by encoding the spatial correlation of colors. Texture features using Local Binary Patterns (LBP) from the image’s red, green, and blue channels have been extracted. LBP is a robust and important texture descriptor that encodes the patterns by comparing each pixel with its neighbors. The combination of these features achieves the highest results and improves the proposed methodology to differentiate between multiple rice plant leaf diseases. Table 7 presents the experimental results of the fusion method according to precision, recall, F1-score, specificity, MCC, and accuracy. The last column in this table indicates the average accuracy for each level. The experimental results are stable from 5 levels and converge for higher levels, as shown in Fig. 9. Therefore, five levels are selected for computation. Fig. 10 refers to the first dataset’s confusion matrix for five folds.

**Table 3:** Experimental results of texture features extraction methods on the first dataset.

		Disease Type				
FE method	Metric (%)	BLB	BL	BS	TU	Overall Accuracy (%)
WLD	Precision	47.66	39.86	44.10	32.98	42.114
	Recall	55.69	36.06	52.06	24.63	
	F1-Score	51.2	38	47.8	28.2	
	Specificity	79.60	79.70	78	83.33	
	MCC	33.60	16.19	22.39	8.83	
	Accuracy	73.62	70.42	71.52	68.66	
GLCM	Precision	69.93	55.54	44.99	68.98	59.046
	Recall	53.37	59.25	48.25	75.39	
	F1-Score	60.6	57.2	46.4	71.8	
	Specificity	92.33	84.20	80.22	88.62	
	MCC	50.36	42.54	27.87	62.17	
	Accuracy	82.59	77.97	72.24	85.29	
LPQ	Precision	54.34	50.22	65.28	91.59	65.504
	Recall	50.94	49.13	69.38	92.56	
	F1-Score	52.2	49.6	67.2	92.2	
	Specificity	85.50	83.68	87.67	97.14	
	MCC	37.22	33.08	55.93	89.39	
	Accuracy	76.86	75.05	83.09	96	
HOG	Precision	69.02	67.01	72.91	70.97	70.202
	Recall	59.31	58.19	78	85.31	
	F1-Score	63.8	62.2	75.2	77.6	
	Specificity	91.12	90.45	90.31	88.37	
	MCC	53.17	51.08	66.83	69.59	
	Accuracy	83.17	82.39	87.24	87.61	
LFD	Precision	62.01	65.46	70.97	87.84	71.51
	Recall	63.03	61.94	74.44	86.56	
	F1-Score	62.6	63.8	72.6	87.2	
	Specificity	87.07	89.14	89.81	96	
	MCC	49.95	52.03	63.27	82.97	
	Accuracy	81.08	82.35	85.97	93.64	
LBP	Precision	70.67	67.18	73.25	82.73	73.83
	Recall	73.5	57.25	75.002	89.56	
	F1-Score	72	61.8	74.2	86	
	Specificity	89.83	90.64	90.85	93.77	
	MCC	62.52	50.66	63.35	81.22	
	Accuracy	85.75	82.29	86.89	92.72	
**MCLBP**	Precision	76.12	71.28	75.88	89.69	**78.594**
	Recall	71.38	61.48	86.44	95.13	
	F1-Score	73.6	65.8	80.6	92.4	
	Specificity	92.52	91.75	90.81	96.37	
	MCC	65.31	56.02	74.11	89.9	
	Accuracy	87.24	84.17	89.72	96.06

**Table 4:** Experimental results of color features extraction methods on the first dataset.

		Disease Type				
Technique	Metric (%)	BLB	BL	BS	TU	Overall Accuracy (%)
Block Color Moment	Precision	79.37	72.26	84.16	99.05	84.23
	Recall	76.44	77.75	85.25	93.94	
	F1-Score	77.87	74.89	84.77	96.47	
	Specificity	93.38	90.16	94.64	99.63	
	MCC	70.71	66.20	75.56	95.39	
	Accuracy	89.14	86.97	92.3	98.28	
**Color Correlogram**	Precision	77.14	83.37	94.57	95.52	**87.15**
	Recall	86.43	81.31	83.62	97.25	
	F1-Score	81.49	82.32	88.72	96.37	
	Specificity	91.41	94.58	98.36	94.48	
	MCC	75.07	76.54	85.58	95.16	
	Accuracy	90.17	91.26	94.7	98.17	
Color Histogram	Precision	80.71	73.46	84.49	87.46	82.3
	Recall	75.7	72.7	84.88	92.8	
	F1-Score	78.6	74.2	83.8	88.8	
	Specificity	94.04	91.92	94.25	93.8	
	MCC	72.6	62.87	80.4	86.19	
	Accuracy	89.81	88.13	90.39	94.69

**Table 5:** Experimental results of ML with LBP method on the first dataset.

		Disease Type				
Number of Levels	Metric (%)	BLB	BL	BS	TU	Overall Accuracy (%)
1 Level	Precision	70.67	67.18	73.25	82.73	73.83
	Recall	73.5	57.25	75.002	89.56	
	F1-Score	72	61.8	74.2	86	
	Specificity	89.83	90.64	90.85	93.77	
	MCC	62.52	50.66	63.35	81.22	
	Accuracy	85.75	82.29	86.89	92.72	
2 Levels	Precision	73.80	67.87	74.28	77.22	73.83
	Recall	71.25	58.81	75.19	88.94	
	F1-Score	72.4	63	74.6	83	
	Specificity	92.52	91.75	90.81	96.37	
	MCC	65.31	56.02	74.11	89.9	
	Accuracy	86.42	82.75	87.27	90.66	
3 Levels	Precision	78.71	72.46	85.49	87.24	81.014
	Recall	76.13	71.75	83.88	92.31	
	F1-Score	77.2	72.2	84.8	89.8	
	Specificity	93.04	90.92	95.25	95.48	
	MCC	69.98	62.87	79.64	86.19	
	Accuracy	88.81	86.13	92.39	94.69	
4 Levels	Precision	79.348	82.192	85.436	95.432	85.578
	Recall	82.624	79.434	83.934	96.306	
	F1-Score	81	80.4	84.8	96	
	Specificity	92.81	94.27	95.23	98.46	
	MCC	74.45	74.56	79.64	94.48	
	Accuracy	90.298	90.534	92.404	97.922	
5 Levels	Precision	88.344	88.824	92.202	98.048	91.828
	Recall	89.872	89.06	90.81	97.558	
	F1-Score	89	88.8	91.8	97.8	
	Specificity	96.04	96.27	97.44	99.35	
	MCC	85.43	85.25	88.7	97.07	
	Accuracy	94.5	94.468	95.782	98.888	
6 Levels	Precision	92.096	93.314	95.894	99.052	95.076
	Recall	93.124	94.622	94.374	98.182	
	F1-Score	92.6	93.8	95.2	98.4	
	Specificity	97.37	97.73	98.65	99.69	
	MCC	90.21	91.93	93.52	98.16	
	Accuracy	96.328	96.938	97.58	99.314	
**7 Levels**	Precision	97.64	97.47	98.19	98.99	**98.064**
	Recall	98.06	98.25	97.56	98.38	
	F1-Score	97.8	98	97.8	98.6	
	Specificity	99.20	99.14	99.39	99.67	
	MCC	97.14	97.14	97.17	98.24	
	Accuracy	98.92	98.92	98.94	99.35

**Table 6:** Experimental results of ML with MCLBP method on the first dataset.

		Disease Type				
Number of Levels	Metric (%)	BLB	BL	BS	TU	Overall Accuracy (%)
1 Level	Precision	76.12	71.28	75.88	89.69	78.594
	Recall	71.38	61.48	86.44	95.13	
	F1-Score	73.6	65.8	80.6	92.4	
	Specificity	92.52	91.75	90.81	96.37	
	MCC	65.31	56.02	74.11	89.9	
	Accuracy	87.24	84.17	89.72	96.06	
2 Levels	Precision	80.97	80.66	87.56	96.49	86.422
	Recall	84.56	76.38	88.75	96.002	
	F1-Score	82.8	78.6	88.2	96.4	
	Specificity	93.37	93.89	95.79	98.83	
	MCC	76.83	71.58	84.16	94.99	
	Accuracy	91.17	89.46	94.03	98.13	
3 Levels	Precision	91.35	91.99	95.03	99.69	94.484
	Recall	94.19	89.5	95.32	98.94	
	F1-Score	92.6	90.8	95.4	99.6	
	Specificity	97.02	97.39	98.33	99.89	
	MCC	90.29	87.70	93.55	99.08	
	Accuracy	96.31	95.42	97.58	99.66	
4 Levels	Precision	97.88	96.72	98.75	99.69	98.252
	Recall	97.88	97.37	98.37	99.38	
	F1-Score	97.8	97	98.2	99.8	
	Specificity	99.29	98.89	99.58	99.89	
	MCC	97.17	96.06	98.08	99.37	
	Accuracy	98.94	98.52	99.28	99.77	
**5 Levels**	Precision	98.89	98.33	99.43	99.69	**99.078**
	Recall	99.19	99.19	98.57	99.38	
	F1-Score	99	98.8	99.2	99.8	
	Specificity	99.62	99.43	99.81	99.89	
	MCC	98.71	98.34	98.66	99.37	
	Accuracy	99.52	99.38	99.5	99.77

**Table 7:** Experimental results of fusion between ML, MCLBP, and color correlogram methods on the first dataset.

		Disease Type				
Number of Levels	Metric (%)	BLB	BL	BS	TU	Overall Accuracy (%)
1 Level	Precision	90.47	86.55	93.49	99.06	92.34
	Recall	89.56	88.56	92.02	99.18	
	F1-Score	90	87.52	92.76	99.12	
	Specificity	96.85	95.39	97.85	99.68	
	MCC	86.71	83.33	90.38	98.83	
	Accuracy	95.03	93.68	96.40	99.56	
2 Levels	Precision	95.32	91.60	96.45	99.93	95.76
	Recall	92.93	95.68	94.93	99.50	
	F1-Score	94.10	93.58	95.68	99.71	
	Specificity	98.47	97.06	98.83	99.98	
	MCC	99.22	91.43	94.27	99.62	
	Accuracy	97.09	96.71	97.85	99.85	
3 Levels	Precision	98.55	97.24	99	99.9	98.68
	Recall	97.5	98.5	99	99.69	
	F1-Score	98.01	97.89	99	99.42	
	Specificity	99.52	99.06	99.66	99.9	
	MCC	97.37	97.19	98.67	99.79	
	Accuracy	99.01	98.93	99.5	99.92	
4 Levels	Precision	99.13	98.02	99.64	99.9	99.20
	Recall	98.81	99.37	99.06	99.56	
	F1-Score	98.97	98.72	99.34	99.78	
	Specificity	99.71	99.35	99.87	99.9	
	MCC	98.62	98.30	99.12	99.70	
	Accuracy	99.48	99.35	99.67	99.88	
**5 Levels**	Precision	99.07	98.69	99.37	99.94	**99.53**
	Recall	99.31	99.44	98.81	99.50	
	F1-Score	99.19	99.06	99.09	99.72	
	Specificity	99.67	99.56	99.79	99.98	
	MCC	98.92	98.75	98.79	99.62	
	Accuracy	99.59	99.53	99.55	99.86	

#### Using the DS2

**Fig. 11:**
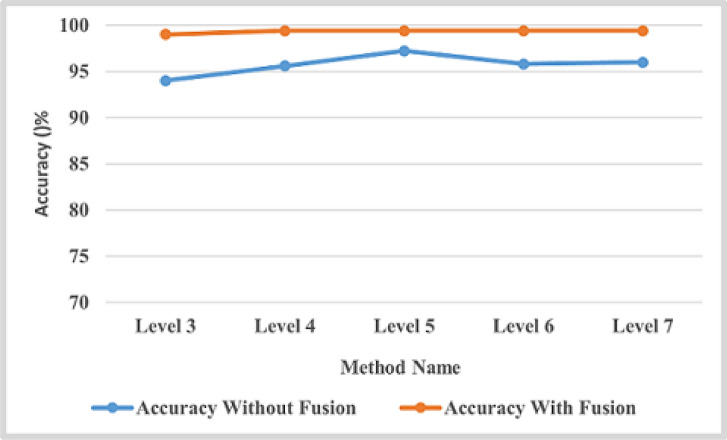
Overall accuracy of each level for the second dataset in case of without and with features fusion.

**Fig. 12:**
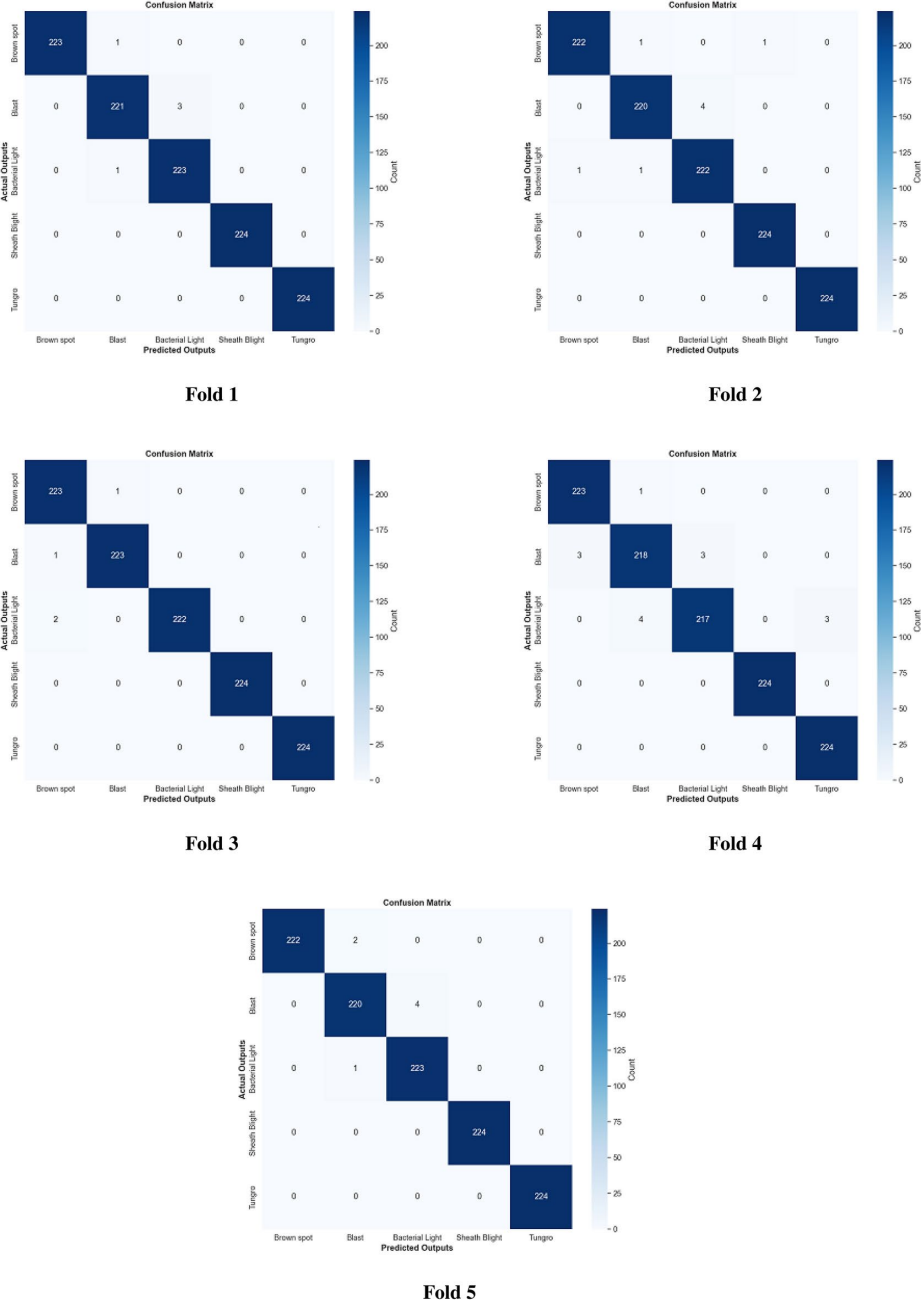
MCLBP ML (5 Levels) confusion matrix for five folds on the second dataset.

This section applied the proposed framework to the second dataset to achieve the generalization concept and validate our work. From the results obtained on the first dataset, we realized that MCLP, in combination with ML representation, outperforms other FE methods. Therefore, the second dataset validates system performance with this combination (i.e., MCLBP with ML). ML representation has been tested on different levels, from 3 to 7. The experimental results before and after fusion method have been computed as precision, recall, F1-score, accuracy, specificity, and MCC for each class, as indicated in Table 8. The overall accuracy for each number of levels has been computed and shown in the last column in Table 8. After that the fusion between color features and multi-level MCLBP achieves highest results, Table 9 show the experimental results of fusion between ML, MCLBP, and color correlogram methods on the second dataset. Figure 11 indicates that, in the case of the second dataset before fusion method, the optimal number of levels is 5, which gives the highest accuracy value, and after that, the results of the next levels decrease, and after applying fusion method, the optimal number of levels is 4. Fig. 12 refers to the confusion matrix for five folds on the second dataset.

**Table 8:** Experimental results of ML with MCLBP method on the second dataset.

		Disease Type					
Number of Levels	Metric (%)	BS	BL	BLB	SB	TU	Overall Accuracy (%)
3 Levels	Precision	93.2	88.6	95	98.2	98.4	94
	Recall	87.9	93.8	95.6	97.4	97.2	
	F1-Score	91.2	91	95.2	97.8	97.8	
	Specificity	98.41	96.98	98.75	99.55	99.57	
	MCC	89.02	88.75	94.10	97.32	97.37	
	Accuracy	96	96	97.6	98.8	98.8	
4 Levels	Precision	95	92.2	95.8	98.2	99	95.6
	Recall	93	94.8	95.8	98	98.6	
	F1-Score	94	93.4	95.8	98.4	99	
	Specificity	98.57	97.97	98.95	99.64	99.84	
	MCC	92.42	91.74	94.87	97.99	98.82	
	Accuracy	97.58	97.32	98.35	99.35	99.62	
5 Levels	Precision	95.8	95.4	96.4	98.4	99	97.22
	Recall	96	95.2	96.8	98.4	98.6	
	F1-Score	95.6	95.4	96.6	98.6	98.8	
	Specificity	98.95	98.90	99.06	99.62	99.84	
	MCC	94.81	94.26	95.77	98.27	98.82	
	Accuracy	97.8	97.6	98	99	99	
6 Levels	Precision	94.6	92.8	95	98.4	98.6	95.8
	Recall	92.8	93.4	96.6	98	98.4	
	F1-Score	93.6	93	95.8	98.2	98.8	
	Specificity	98.59	98.16	98.73	99.59	99.73	
	MCC	91.93	91.20	94.69	97.88	98.43	
	Accuracy	97	96.6	97.6	98.8	99	
**7 Levels**	Precision	94	93.6	94.4	98.4	98.8	**96**
	Recall	94.2	93.4	95.6	98	98	
	F1-Score	94	93.6	95.2	98	98.6	
	Specificity	98.48	98.39	98.63	99.55	99.75	
	MCC	92.53	91.74	93.84	97.71	98.32	
	Accuracy	97	97	97.4	98.8	99

**Table 9:** Experimental results of fusion between ML, MCLBP, and color correlogram methods on the second dataset

		Disease Type					
Number of Levels	Metric (%)	BS	BL	BLB	SB	TU	Overall Accuracy (%)
1 Level	Precision	96.79	98.09	98.9	98.86	98.86	98
	Recall	98.92	96.69	95.8	99.9	99.9	
	F1-Score	97.84	97.38	97.32	99.42	99.42	
	Specificity	99.17	99.53	99.73	99.71	99.71	
	MCC	97.30	96.74	96.69	99.29	99.28	
	Accuracy	99.12	98.96	98.94	99.76	99.76	
2 Levels	Precision	98.85	98.2	99.3	99.8	99.5	98.5
	Recall	99.64	97.76	97.67	99.5	99.5	
	F1-Score	99.24	97.98	98.33	99.5	99.5	
	Specificity	99.5	99.6	99.7	99.89	99.5	
	MCC	99	97.48	97.92	99.39	99.5	
	Accuracy	99.69	99.19	99.33	99.80	99.8	
3 Levels	Precision	99	98.2	98.48	99.3	99.5	99
	Recall	98.83	97.85	98.3	99.5	99.5	
	F1-Score	98.93	98	98.38	99.64	99.5	
	Specificity	99.75	99.54	99.62	99.82	99.5	
	MCC	98.66	97.54	97.99	99.56	99.5	
	Accuracy	99.57	99.2	99.35	99.85	99.5	
**4 Levels**	Precision	99.37	98.83	98.75	98.64	99.5	**99.4**
	Recall	99.37	98.39	98.84	99.9	99.9	
	F1-Score	99.37	98.61	98.79	99.82	99.5	
	Specificity	99.84	99.71	99.68	99.9	99.9	
	MCC	99.22	98.26	98.49	99.77	99.5	
	Accuracy	99.74	99.44	99.51	99.92	99.5	

#### Using the DS3

**Fig. 13:**
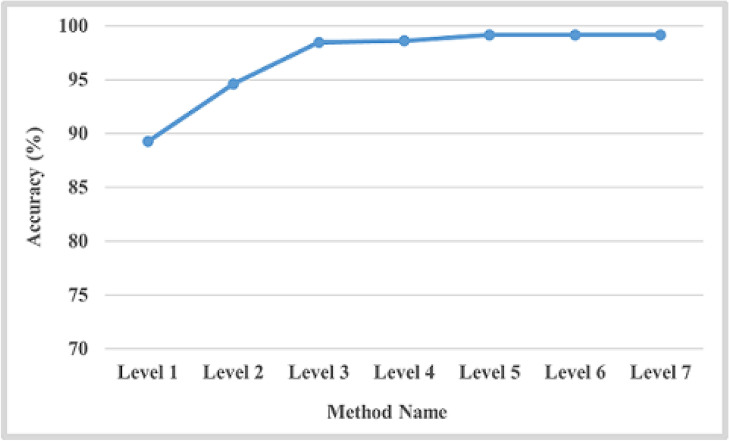
The overall accuracy of each level for the third dataset in the case of features fusion.

**Fig. 14:**
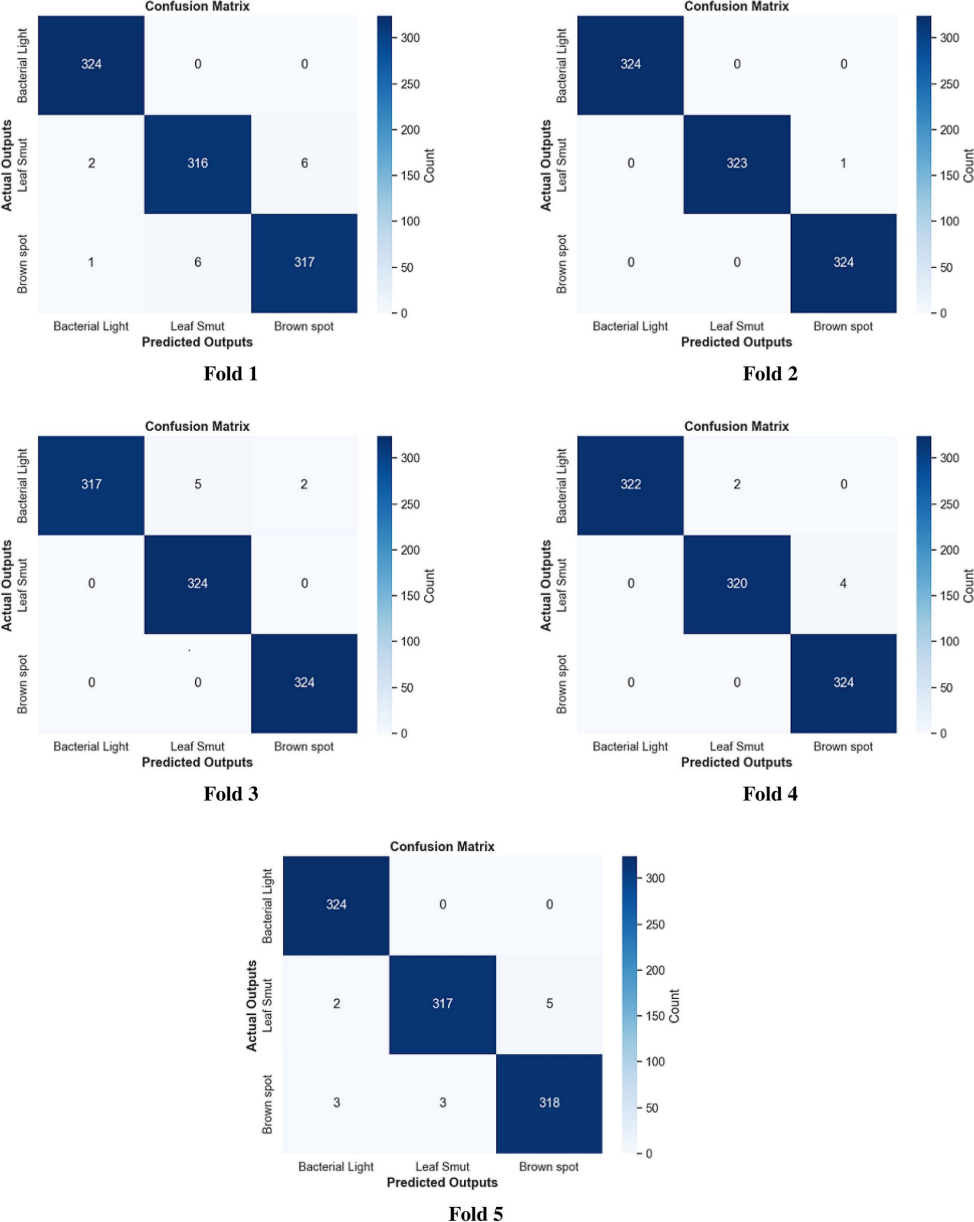
MCLBP ML (5 Levels) confusion matrix for five folds on the third dataset.

This section applied the proposed framework to the third dataset to achieve the generalization concept and validate our work. We realized from the results obtained on the first and second datasets that the fusion method between color features and color texture features outperforms other FE methods. Therefore, the third dataset validates system performance with this combination (i.e., MCLBP with ML). ML representation has been tested on different levels, from 1 to 5. After the fusion method, the experimental results have been computed as precision, recall, F1-score, accuracy, specificity, and MCC for each class, as indicated in Table 10. The experimental results indicate that the results remain constant from level 5 as shown in Figure 13. Figure 14 presents the confusion matrices for five fold cross validation.

**Table 10:** Experimental results of fusion between ML, MCLBP, and color correlogram methods on the third dataset.

		Disease Type			
Number of Levels	Metric (%)	BLB	LS	BS	Overall Accuracy (%)
1 Level	Precision	90.14	85.03	92.72	89.24
	Recall	89.2	86.91	91.6	
	F1-Score	89.65	85.9	92.14	
	Specificity	95.12	92.34	96.38	
	MCC	84.54	78.84	88.27	
	Accuracy	93.14	90.53	94.79	
2 Levels	Precision	95.24	91.79	96.98	94.59
	Recall	93.52	95.43	94.81	
	F1-Score	94.35	93.55	95.87	
	Specificity	97.65	95.71	98.51	
	MCC	91.6	90.3	93.87	
	Accuracy	96.27	95.61	97.28	
3 Levels	Precision	98.63	97.55	99.2	98.45
	Recall	97.9	98.51	98.95	
	F1-Score	98.26	98.03	99.07	
	Specificity	99.32	98.76	99.6	
	MCC	97.4	97.2	98.61	
	Accuracy	98.84	98.68	99.38	
4 Levels	Precision	99.4	98.16	99.32	98.6
	Recall	98.76	98.95	99.2	
	F1-Score	99	98.5	99.25	
	Specificity	99.7	99.07	99.66	
	MCC	98.65	97.83	98.89	
	Accuracy	99.4	99	99.5	
**5 Levels**	Precision	99.51	99.01	98.89	**99.14**
	Recall	99.44	98.76	99.19	
	F1-Score	99.47	98.88	99.04	
	Specificity	99.75	99.51	99.44	
	MCC	99.21	98.33	98.56	
	Accuracy	99.65	99.26	99.36	

### A comparison of different classification methods

Classification is the final step in the proposed rice plant disease recognition framework. This step is essential for predicting the class of a new image. To achieve more accurate and robust predictions, a various classifiers have been evaluated and implemented. These include some traditional machine learning algorithms such as Naive Bayes^100^ which depends on probabilistic principles, k nearest neighbor (KNN)^101^ which is efficient in non linear data, Decision tree^102^ which is non-parametric supervised learning.

In addition to these, multiple boosting family methods have been applied to enhance classification performance. This includes AdaBoostM2^103^ which enhances weak learners, Bag Ensemble^104^ that decreases the variance by averaging multiple models, Subspace Ensemble^105^ which depends on random subspace of features, RusBoost Ensemble^106^ that used to handle imbalanced dataset.

Furthermore, deep learning classifiers such as artificial neural network (ANN) and multi layer perceptron (MLP) which are able to capture complex patterns in high-dimensional data, and also SVM^107^ with three kernels: RBF, polynomial, and linear.

Table 11 presents the evaluation of the proposed system, which is based on features fusion between color features with color correlogram and color texture features with MCLBP that depended on Multi-Level representation, denoted that one-against-all support vector machine (SVM) with linear kernel has highest experimental results than other classifiers. Every dataset has special case for levels ccount as for DS1, the number of levels that achieved highest results is 5 levels with 99.53% accuracy, for DS2, 4 levels with 99.4% accuracy, and for DS3 5 levels has highest accuracy with 99.14%.

**Table 11:** The experimental results of the proposed methodology with other used classifiers tested on three datasets

Datasets	Classifier	Precision (%)	Recall (%)	F1-Score (%)	Specificity (%)	MCC (%)	Accuracy (%)
DS1	KNN	90.8	90.8	90.7	87.7	96.5	95.4
	Naive Bayes	72.5	73.42	72.65	70.5	92.7	87.5
	Decision Tree	96.3	96.4	94.9	95.36	96.8	97.2
	Ensemble Classifier (Bag)	92.59	92.31	92.3	99.15	92	98.47
	Ensemble Classifier (Subspace)	80.76	76.23	76.45	70.5	90.73	88.11
	Ensemble Classifier (RusBoost)	82.5	79.5	78.8	75.2	96.5	93.09
	Ensemble Classifier (AdaBoostM2)	84.62	97.41	90.74	98.2	89.8	98.2
	Multi Layer Perceptron	92.4	92.5	92.45	91.2	96.8	96.5
	Artificial Neural Network	63.2	60.3	90.3	60.5	88.5	74.5
	SVM (RBF kernel)	93.9	91.9	92.3	90.2	95.2	93.67
	SVM (Polynomial kernel)	87.28	86.37	86.03	98.49	85.2	97.28
	**SVM (linear kernel)**	**99.6**	**99.4**	**99.4**	**99**	**99.2**	**99.53**
DS2	KNN	93.16	93.08	93.01	91.38	98.27	97.23
	Naive Bayes	70.3	70.42	70.65	68.5	90.7	85.5
	Decision Tree	95.5	95.4	95.4	94.36	96.2	96.16
	Ensemble Classifier (Bag)	84.92	97.41	90.74	98.08	89.9	98.01
	Ensemble Classifier (Subspace)	72.96	70.3	70	63.86	92.57	88.12
	Ensemble Classifier (RusBoost)	79	77.73	77.68	72.64	94.43	91.09
	Ensemble Classifier (AdaBoostM2)	85.03	95.42	89.93	98.13	88.9	97.86
	Multi Layer Perceptron	93.76	93.76	93.72	92.2	98.44	97.50
	Artificial Neural Network	70.9	62.7	64.8	94.7	93.2	75.8
	SVM (RBF kernel)	92.96	89.48	89.84	88.31	97.37	95.79
	SVM (Polynomial kernel)	81.22	96.32	88.13	97.53	87.2	97.41
	**SVM (linear kernel)**	**98.8**	**99.2**	**99.2**	**99.15**	**99**	**99.4**
DS3	KNN	90.87	90.78	90.76	95.39	86.23	93.85
	Naive Bayes	69.5	68.88	70.5	66.5	87.7	82.5
	Decision Tree	94.5	93.8	94.5	95.2	95.4	95.4
	Ensemble Classifier (Bag)	87.74	86.73	86.5	98.53	85	97.4
	Ensemble Classifier (Subspace)	73.9	71.2	71.33	60.2	85.56	80.88
	Ensemble Classifier (RusBoost)	80.26	78.78	78.95	89.39	68.93	85.85
	Ensemble Classifier (AdaBoostM2)	88.2	86.85	87.2	98.7	85.2	97.8
	Multi Layer Perceptron	86.85	86.6	86.5	93.3	80.06	91.07
	Artificial Neural Network	67.6	63.8	64.3	95	93.5	78.7
	SVM (RBF kernel)	93.67	92.18	92.33	96.08	89.07	96.08
	SVM (Polynomial kernel)	95.5	94.2	94.8	97.3	92.2	97.08
	**SVM (linear kernel)**	**99.13**	**99.14**	**99.2**	**99.56**	**98.7**	**99.42**

### Computational complexity analysis with deep learning models

The efficiency and accuracy of machine learning models have a crucial role in rice plant disease detection and classification. To evaluate the efficiency of the proposed system, it is compared with prominent TensorFlow-based models such as VGG16 ^108^, InceptionV3 ^109^, ResNet50 ^110^, DensNet ^111^, InceptionResNetV2 ^112^. Table 12 indicates the reliability of this proposed system with the TensorFlow models. This comparison concentrates on two key metrics: processing time and accuracy. The accuracy and processing time were computed based on performance evaluation for 10 rice images. VGG16 model achieves 86.55% accuracy with 244 ms processing time, InceptionV3 achieves 88.64% with 134 ms processing time, ResNet50 achieves 69.76% accuracy with 143 ms processing time, DensNet achieves 91.87% accuracy with 187 ms processing time, InceptionResNetV2 achieves 81.56% accuracy with 171 ms processing time, and the proposed system achieves 99.5% accuracy with processing time from $100(\pm 17)ms$ and this according to applying four or five multi-level.

**Table 12:** The average accuracy and processing time compared with some TensorFlow deep learning models.

Model	Processing Time	Accuracy
VGG16	244 ms	86.55%
InceptionV3	134 ms	88.64%
ResNet50	143 ms	69.76%
DensNet	187 ms	91.87%
InceptionResNetV2	171 ms	81.56%
Proposed System	$100(\pm 17)ms$	99.5%

### A Comparison with the state-of-the-art methods

Comparison with the state-of-the-art methods is provided in this section. Table 13 contains the comparative results between the proposed framework and other methods tested on three tested datasets with the state of the art papers according to each disease. As shown in Table 13, the proposed system achieved highest experimental results for each class.Also Table 14 contains the comparative results between the proposed framework and other methods tested on three tested datasets. The comparison of the first dataset works on four types of rice plant diseases: BLB, BL, BS, and TU. The proposed framework achieves 99.53% for accuracy with 5 levels on the first dataset. Also, in Table 13, there are the comparative results between the proposed framework and other methods tested on the second dataset. For the second dataset, the methods don’t work on the same number of classes, and this is because of a limited dataset. Rice leaves diseases in second dataset as BLB, BL, and BS. The proposed framework achieves 99.4% for accuracy with four levels on the second dataset. According to the third dataset, the rice Rice leaves diseases are BLB, BS, and LS. The proposed framework achieves 99.14% for accuracy with five levels on the third dataset.

**Table 13:** Experimental results with other related work based on disease class.

		Metric (%)					
Paper	Disease Type	Precision	Recall	F1-Score	Specificity	MCC	Accuracy
Sethy *et al.*^37^ (2020)	BLB	96.6	98.5	97.5	98.33	94.5	98.75
	BL	99.5	96.5	96.5	98.33	92.4	98.25
	BS	100	98	98.9	99.8	99.5	98.6
	TU	100	99.5	100	100	100	97.8
Rahim *et al.*^113^ (2023)	BLB	100	93	96	95	93	95.5
	BL	100	94	97	94	93.8	96.4
	BS	89	100	94	95	94	95.8
	TU	100	100	100	100	100	100
Sharma *et al.*^114^ (2022)	BLB	98.32	98.65	98.48	99.23	92.4	99.05
	BL	92.85	99.04	95.85	97.82	93.5	98
	BS	99.11	94.53	96.77	99.71	95.4	98.4
	TU	94.94	93.06	94	98.65	96.4	97.45
Saminathan *et al.*^115^ (2023)	BLB	97.85	97.85	97.85	99.25	96.5	98.9
	BL	95.71	97	96.4	98.5	97.4	98.2
	BS	97.85	98.65	98.2	99.28	97.6	99
	TU	98.57	96.5	97.5	99.5	98.2	98.75
Zeng *et al.*^116^ (2023)	BLB	98.1	97	97.55	99.3	96.4	98.67
	BL	94.85	94.58	94.71	98.39	95.7	97.48
	BS	93.62	96.39	94.99	97.64	94.8	97.32
	TU	98.24	96.47	97.34	99.49	96.49	98.81
Asfarian *et al.*^32^ (2013)	BLB	100	88.8	94.12	100	93.77	99
	BL	91.66	96.25	93.9	96.88	91.7	96.7
	BS	95.4	83	88.7	98	84.2	93
	TU	87.27	97.95	92.3	93.32	88.66	94.75
Saha and ahsan *et al.*^34^ (2021)	BLB	88.3	94.5	91.29	92.5	91.7	94.53
	SB	91.4	91.4	91.4	93.2	92.5	94.11
	BL	95.4	83.3	88.94	80.4	81.2	83.33
	BS	95	98.9	96.91	96.5	94.8	98.97
	TU	91.6	91.6	91.6	75.6	76.2	78.57
kumar *et al.*^117^ (2022)	BLB	80	100	88.89	92.85	86.18	94.44
	BS	100	100	100	100	100	100
	LS	100	88.89	94.11	100	89.44	94.4
Hassan *et al.*^118^ (2023)	BLB	98.09	97.16	97.63	99.04	96.5	98.41
	BS	100	96.77	98.36	100	93.4	98.88
	LS	95.71	100	97.81	97.9	91.4	98.57
Rathore *et al.*^119^ (2023)	BLB	85.72	100	92.3	93.75	89.64	95.45
	BS	100	83.33	90.90	100	86.43	93.18
	LS	93.33	100	96.55	96.67	94.98	97.77
Proposed (DS1)	BLB	99.07	99.31	99.19	99.67	98.92	99.59
	BL	98.69	99.44	99.06	99.56	98.75	99.53
	BS	99.37	98.81	99.09	99.79	98.79	99.55
	TU	99.94	99.50	99.72	99.98	99.62	99.86
Proposed (DS2)	BS	99.37	99.37	99.37	99.84	99.22	99.74
	BL	98.83	98.39	98.61	99.71	98.26	99.44
	BLB	98.75	98.84	98.79	99.68	98.49	99.51
	SB	98.64	99.6	99.82	99.9	99.77	99.92
	TU	99.5	99.9	99.5	99.9	99.5	99.5
Proposed (DS3)	BLB	99.51	99.44	99.47	99.75	99.21	99.65
	BS	99.01	98.76	98.88	99.51	98.33	99.26
	LS	98.89	99.19	99.04	99.44	98.56	99.36

**Table 14:** Experimental results with other related work on the two tested datasets.

Datasets	Paper	Class name	Accuracy (%)
DS1	Sethy *et al.* ^37^ (2020)	BLB, BL, BS and TU	98.38
	Ganesan and Chinnappan ^38^ (2022)	BLB, BL, BS and TU	97.67
	Mohapatra *et al.* ^42^ (2022)	BLB, BL, BS and TU	97.47
	Sharma *et al.* ^114^ (2022)	BLB, BL, BS and TU	99.5
	Daniya and Srinivasan ^43^ (2023)	BLB, BL, BS and TU	92.6
	Haruna *et al.* ^44^ (2023)	BLB, BL, BS and TU	91.83
	Rahim *et al.* ^113^ (2023)	BLB, BL, BS and TU	96
	Saminathan *et al.* ^115^ (2023)	BLB, BL, BS and TU	97.62
	Zeng *et al.* ^116^ (2023)	BLB, BL, BS and TU	95.57
	Arya *et al.* ^46^ (2024)	BLB, BL, BS and TU	98.75
	**Proposed Method**	BLB, BL, BS and TU	**99.53**
DS2	Prajapati *et al.* ^31^ (2017)	BLB, BL, and BS	62
	Asfarian *et al.* ^32^ (2013)	BLB, TU BL, and BL	83
	Pinki *et al.* ^33^ (2017)	BLB, BL, and BS	92.06
	Saha and ahsan *et al.* ^34^ (2021)	BS, BL, BL, TU, and SB	92.77
	**Proposed Method**	BS, BL, BLB, TU, and SB	**99.4**
DS3	kumar *et al.* ^117^ (2022)	BLB, BS, LS	98.8
	Hassan *et al.* ^118^ (2023)	BLB, BS, LS	97.9
	Rathore *et al.* ^119^ (2023)	BLB, BS, LS	93.75
	Hossen *et al.* ^120^ (2024)	BLB, BS, LS	98
	Chaudhary *et al.* ^35^ (2024)	BLB, BS, LS	96.7
	Pandi *et al.* ^121^ (2024)	BLB, BS, LS	96.7
	**Proposed Method**	BLB, BS, LS	**99.14**

## Conclusion

Rice diseases threaten global food security and increase every day. It is very significant to maintain rice production. Early detection of rice plant diseases is needed. This paper proposes a new system for detecting and classifying rice plant leaf diseases by fusing different features, including color texture using local binary pattern (LBP) and color features using color correlogram (CC). The proposed system has been validated on three datasets with six classes: BL, BLB, BS, TU, SB, and Image preprocessing, which is applied through data augmentation and enhancement techniques. The features extraction stage is responsible for extracting color features using CC and color texture features using multi-level multi-channel local binary pattern (MCLBP). Finally, Rice image classification is implemented using a one-against-all support vector machine (SVM). To validate the proposed system, it has been compared with the state-of-the-art papers for rice plant leaf disease detection and classification and state-of-the-art machine and deep learning classifiers on three public benchmark datasets to prove the concept of applying the proposed system. The first dataset has four classes: BLB, BL, BS, and TU; the second dataset has three BLB, BL, and BS, and the third dataset has three classes: BLB, BS, and LS. Experimental results indicate that the proposed method achieves an accuracy of 99.53% with five levels on the first dataset, 99.4% with four levels on the second dataset, and 99.14% with five levels on the third dataset with a processing time from $100(\pm 17)ms$. This system achieves the highest accuracy and helps detect and classify rice plant diseases, improving agriculture. Despite the highest and most promising experimental results, the proposed system has room for improvement. Some aspects can be applied in the future, such as various feature selection and reduction methods and deep learning models for rice plant disease classification. The proposed framework will also be tested on other plant leaves.

## Data Availability

This research study was tested using three datasets which are publicly available in: https://data.mendeley.com/datasets/fwcj7stb8r/1. https://www.kaggle.com/datasets/rajeshbhattacharjee/rice-diseases-using-cnn-and-svm. https://data.mendeley.com/datasets/dwtn3c6w6p/1.
